# Interaction of Bartonella henselae with Fibronectin Represents the Molecular Basis for Adhesion to Host Cells

**DOI:** 10.1128/spectrum.00598-22

**Published:** 2022-04-18

**Authors:** Diana J. Vaca, Arno Thibau, Matthias S. Leisegang, Johan Malmström, Dirk Linke, Johannes A. Eble, Wibke Ballhorn, Martin Schaller, Lotta Happonen, Volkhard A. J. Kempf

**Affiliations:** a Institute of Medical Microbiology and Infection Control, Goethe University Frankfurt, Frankfurt am Main, Germany; b Institute for Cardiovascular Physiology, Goethe University Frankfurt, Frankfurt am Main, Germany; c Division of Infection Medicine, Lund University, Lund, Sweden; d Department of Biosciences, University of Oslo, Oslo, Norway; e Institute for Physiological Chemistry and Pathobiochemistry, University of Münster, Münster, Germany; f Department of Dermatology, Eberhard Karls-University of Tübingen, Tübingen, Germany; Griffith University

**Keywords:** trimeric autotransporter adhesin, *Bartonella* adhesin A, extracellular matrix, bacterium-host interaction, CRISPR-Cas, cross-linking mass spectrometry

## Abstract

Bacterial adhesion to the host is the most decisive step in infections. Trimeric autotransporter adhesins (TAA) are important pathogenicity factors of Gram-negative bacteria. The prototypic TAA *Bartonella* adhesin A (BadA) from human-pathogenic Bartonella henselae mediates bacterial adherence to endothelial cells (ECs) and extracellular matrix proteins. Here, we determined the interaction between BadA and fibronectin (Fn) to be essential for bacterial host cell adhesion. BadA interactions occur within the heparin-binding domains of Fn. The exact binding sites were revealed by mass spectrometry analysis of chemically cross-linked whole-cell bacteria and Fn. Specific BadA interactions with defined Fn regions represent the molecular basis for bacterial adhesion to ECs and these data were confirmed by BadA-deficient bacteria and CRISPR-Cas knockout Fn host cells. Interactions between TAAs and the extracellular matrix might represent the key step for adherence of human-pathogenic Gram-negative bacteria to the host.

**IMPORTANCE** Deciphering the mechanisms of bacterial host cell adhesion is a clue for preventing infections. We describe the underestimated role that the extracellular matrix protein fibronectin plays in the adhesion of human-pathogenic Bartonella henselae to host cells. Fibronectin-binding is mediated by a trimeric autotransporter adhesin (TAA) also present in many other human-pathogenic Gram-negative bacteria. We demonstrate that both TAA and host-fibronectin contribute significantly to bacterial adhesion, and we present the exact sequence of interacting amino acids from both proteins. Our work shows the domain-specific pattern of interaction between the TAA and fibronectin to adhere to host cells and opens the perspective to fight bacterial infections by inhibiting bacterial adhesion which represents generally the first step in infections.

## INTRODUCTION

Bacterial adhesion to the host represents the first and decisive step in colonization and infection. Bacteria express numerous adhesins to target specific molecular components of the host surface. The wide distribution of extracellular matrix (ECM) proteins in connective tissue and basement membranes make them an attractive host partner for bacterial binding. Fibronectin (Fn), a component of the ECM, comes in different isoforms: (i) plasma Fn present in body fluids and (ii) cellular Fn present on cell surfaces connecting the cells with the pericellular environment and ECM components, e.g., collagen and laminin (1). Fn has been described as an important target for many bacterial adhesins, so-called Fn-binding proteins (FnBPs) which mediate host-adhesion and bacterial virulence (2–4). Inhibition of bacterial adhesion by the use of “anti-ligands” might represent a promising anti-infective strategy, and such innovative therapeutic concepts have been explored experimentally using, e.g., antagonists for FimH from uropathogenic Escherichia coli to treat urinary tract infections (5, 6).

In Gram-negative bacteria, the family of trimeric autotransporter adhesins (TAA) is a major determinant for infection. Consequently, TAA deletion results in reduced virulence and loss of binding to ECM proteins, shown, e.g., for Yersinia enterocolitica, Bartonella henselae, and Acinetobacter baumannii (7–9). TAAs are expressed on the bacterial surface and are characterized by a conserved “membrane anchor domain” at the C-terminus, and a modular “passenger domain” which includes a “head” and an often highly repetitive “neck/stalk” structures at the N-terminus. Variations of this domain assembly are frequently observed (10, 11).

*Bartonella* adhesin A (BadA) is one of the longest TAAs characterized so far, consisting of 3,926 amino acids resulting in a molecular weight of about 417 kDa per monomer (1,251 kDa in its trimeric structure). This prototypical TAA consists of different (sub-)domains conferring domain-specific biological functions for host cell adherence and angiogenesis (12, 13). BadA is a major pathogenicity factor of B. henselae, the causative agent of cat scratch disease and vasculoproliferative diseases in humans (e.g., bacillary angiomatosis). The expression of BadA is crucial for HIF-1 driven angiogenic reprogramming, biofilm formation, and bacterial binding to endothelial cells (ECs) and ECM proteins (7, 14). However, the exact BadA binding mechanism for host cell adhesion is still unknown.

Advanced technologies to broadly analyze protein-protein interactions offer great potential for the description of host-pathogen interactions. By using chemical cross-linking followed by mass spectrometry analysis (XL-MS), novel interspecies protein-protein interactions have been described and provide a deeper understanding of their role in bacterial pathogenicity (15–17). In this work, BadA expressing and BadA-deficient B. henselae, CRISPR-Cas knockout ECs, bacterial binding assays, and XL-MS were used to determine the exact molecular basis and the interaction interfaces for the binding of B. henselae to ECs via BadA-Fn bridging. Our findings provide a deeper understanding of the initial adhesion events in infections which represent the basis for the inhibition of bacterial binding to the host and guide a way to future anti-infective therapeutic strategies for other human pathogens.

## RESULTS

### Expression of BadA is crucial for binding of B. henselae to human Fn.

**FIG 1:**
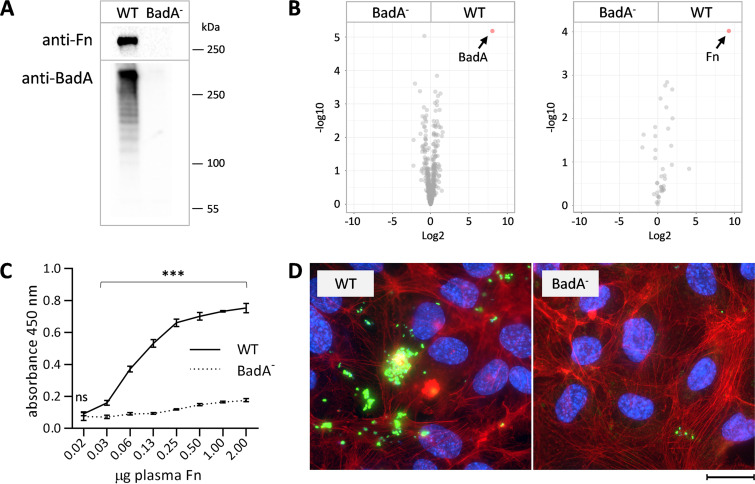
Expression of BadA is crucial for B. henselae binding to Fn. (A) Fn bound to B. henselae WT and BadA^-^. BadA (417 kDa monomer) and Fn (260 kDa monomer) proteins (resolving gel) are shown. For BadA protein, WT strain shows a ladder-like structure resulting from incomplete denaturation of the trimeric BadA. (B) Volcano plot from mass spectrometry data demonstrating magnitude and significance of differing proteins identified in B. henselae WT and BadA^-^ grown on human Columbia blood agar (hCBA). Arrows indicate proteins differing by log_2_ > 2-fold and *P*-value of 0.01 for the bacterial (left) and human (right) proteome. (C) Binding of B. henselae WT and BadA^-^ (1.5 × 10^8^ cells) to increasing amounts of immobilized Fn. Bacteria were detected using anti-B. henselae antibodies. The mean and SD of quadruplicates are depicted. Statistical significance was determined using a two-way ANOVA with Šídák’s multiple-comparison test between WT and BadA^-^ (ns: not significant, *** *P* < 0.0001). (D) Immunofluorescence microscopy of endothelial cells infected with B. henselae WT and BadA^-^ (bacteria: green; nuclei: blue, beta-actin: red. Scale bar: 30 μm).

The BadA-dependent interaction with human Fn was first confirmed by cultivation of B. henselae wild type (WT, expressing BadA) *and* B. henselae BadA^-^ (deficient in BadA expression) on agar plates containing defibrinated human blood (human Columbia blood agar, hCBA). Bacteria-bound Fn was detected via Western blotting (Fig. 1A) and via label-free data-independent acquisition MS-based proteomics (DIA-MS). The MS data were stringently filtered to identify and quantify proteins differing in abundance by log_2_ > 2-fold and by an adjusted *P*-value of 0.01 between the WT and the BadA^-^ strains. We identified Fn as the only protein in human blood that bound B. henselae in a BadA-dependent manner (Fig. 1B). ELISA-based titrations of Fn with B. henselae showed a concentration-dependent adherence for B. henselae WT, with a saturation of Fn reached at 1.0 μg per well, and a strongly reduced binding of B. henselae BadA^-^ (Fig. 1C). The interaction of BadA with Fn was also visible in immunoelectron microscopy of bacteria pre-adsorbed with soluble Fn, indicating Fn deposition at the surface of the WT strain with nearly no Fn presence on the surface of B. henselae BadA^-^ (Fig. S1A). Additionally, we confirmed that adherence of B. henselae to ECs depends strictly on BadA expression (Fig. 1D). Furthermore, Fn known to be produced by ECs was prominently localized in the ECM environment (Fig. S1B), making it a favorable candidate as a primary host interaction partner for bacterial BadA.

### Presence of Fn on ECs determines B. henselae adhesion.

**FIG 2:**
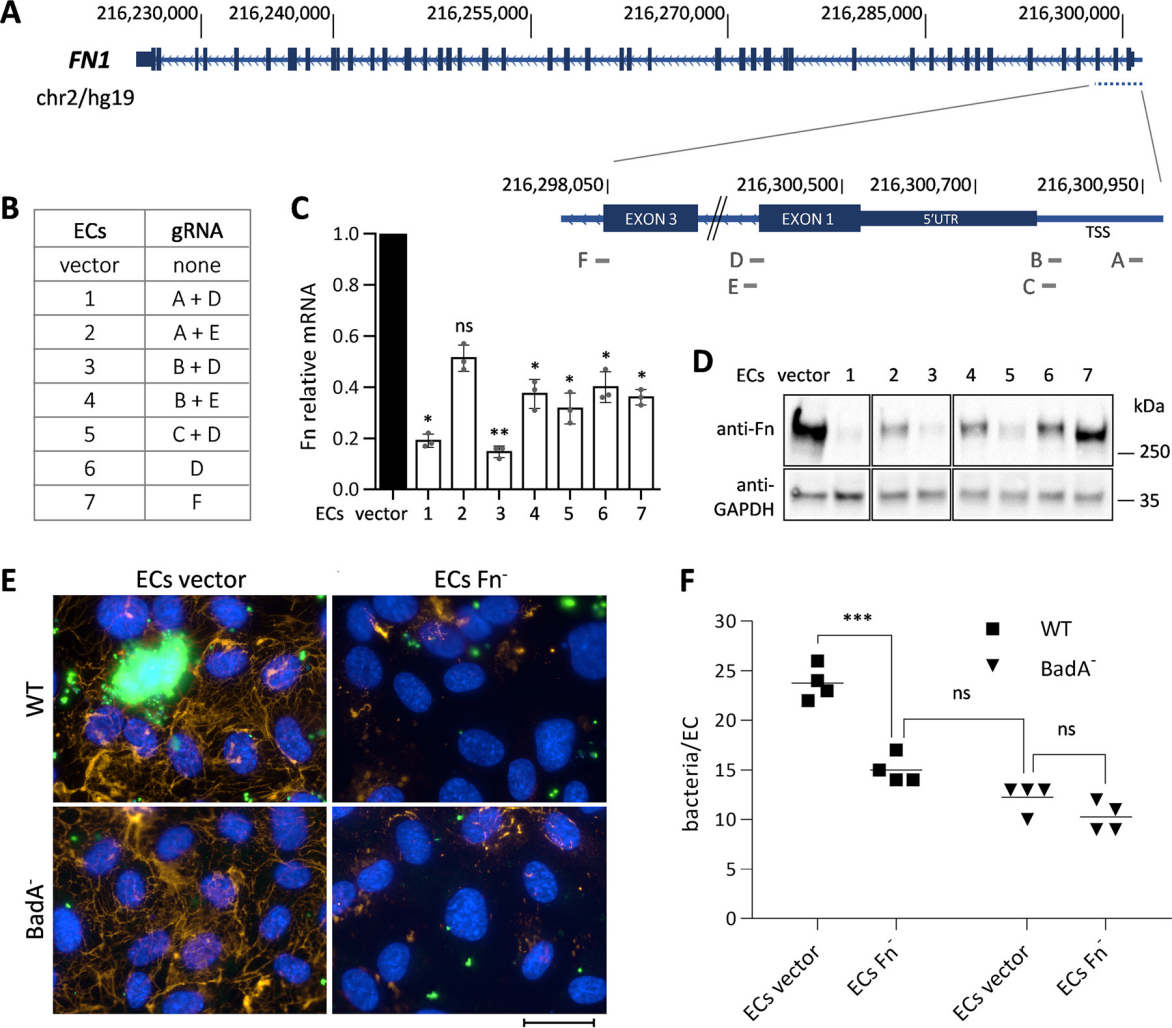
Presence of Fn on ECs determines B. henselae adhesion. (A) Schematic draft of the *FN1* gene and targeted areas for guide RNAs (gRNA) design, including 5′ untranslated region (UTR), transcription starting site (TSS), introns 1 and 3 (left to the respective exons). (B) Combinations of gRNA applied for the generation of Fn-knockout ECs (vector: empty [WT phenotype]). (C) Relative quantification of normalized Fn mRNA levels (housekeeping gene for calibration: *beta-actin*). The mean and SD of triplicates are depicted. Statistical significance was determined using one-way ANOVA with Dunnett’s multiple-comparison test (ns: not significant; * *P* < 0.0096; ** *P* = 0.0007). (D) Analysis of Fn protein expression in ECs via Western blotting (loading control: GAPDH). (E) Immunofluorescence microscopy of ECs vector (control) and ECs Fn^-^ (Fn-knockout ECs 1) infected with B. henselae WT or BadA^-^. Infection time: 60 min (Fn: orange, bacteria: green, nuclei: blue. Scale bar: 30 μm). (F) Absolute quantification of bacterial binding to ECs via qPCR (bacteria: *glyA* gene equivalents; ECs: *hmbs* gene equivalents). The mean and SD of quadruplicates are depicted. Statistical significance was determined using two-way ANOVA with Šídák’s multiple-comparison test (ns: not significant; *** *P* < 0.0001).

Loss-of-function experiments were performed to test whether Fn might contribute to B. henselae adhesion to ECs. For this, Fn knockout ECs were generated applying single or dual guide RNA (gRNA) directed lentiviral CRISPR-Cas9 targeting the *FN1* gene (Fig. 2A). Different combinations using dual gRNA (ECs 1–5) or single gRNA (ECs 6, 7) were tested for efficiency and an empty vector was used for maintaining the WT phenotype (ECs vector) (Fig. 2B). The dual gRNA approach targeting the transcription start site (TSS, gRNA A) or 5′ UTR (gRNA B) and the intron 1 region (gRNA D) lead to successful Fn removal as demonstrated via mRNA expression (RT-qPCR) and protein presence (Western blot), with the best efficiency observed in ECs 1 and 3 (Fig. 2C and D). Fn-knockout was also corroborated by immunofluorescence (IF) staining (Fig. S2A). As Fn acts as a scaffold for deposition of other ECM proteins (18), and BadA has proven to bind also collagen and laminin (19), we evaluated the pericellular collagen V and laminin arrangement via IF, which appeared to be unaffected in Fn-knockout ECs (Fig. S2B). From this, the herein generated Fn-knockout ECs (ECs Fn^-^) were suggested to represent a valuable tool for functional analysis of Fn-dependent binding of B. henselae to ECs and were used in bacterial adherence assays (ECs 1) (Fig. 2E and F). Adhesion was assessed 60 min after infection, as only a neglectable number of bacteria is expected to invade ECs at this time point (20). Evaluation was performed via immunofluorescence microscopy (Fig. 2E), and via qPCR absolute quantification of adherent bacteria per EC (using bacterial *glyA* and human *hmbs* gene equivalents) (Fig. 2F). A significant reduction in host cell adhesion of B. henselae WT was observed for ECs Fn^-^ compared to ECs vector control, proving the crucial role of Fn in B. henselae adherence. In line with this observation, no significant difference was observed between B. henselae BadA^-^ adhesion to ECs Fn^-^ or ECs vector (Fig. 2F). Although BadA-Fn interaction seems to be of major importance for B. henselae adhesion to ECs, bacterial binding in the absence of both proteins (B. henselae BadA^-^ infecting ECs Fn^-^) was not completely abolished (binding reduction of approximately 60%), suggesting that additional binding mechanisms are involved in host cell adhesion, like BadA interaction with other ECM proteins.

### Functional analysis of the BadA binding site in Fn.

**FIG 3:**
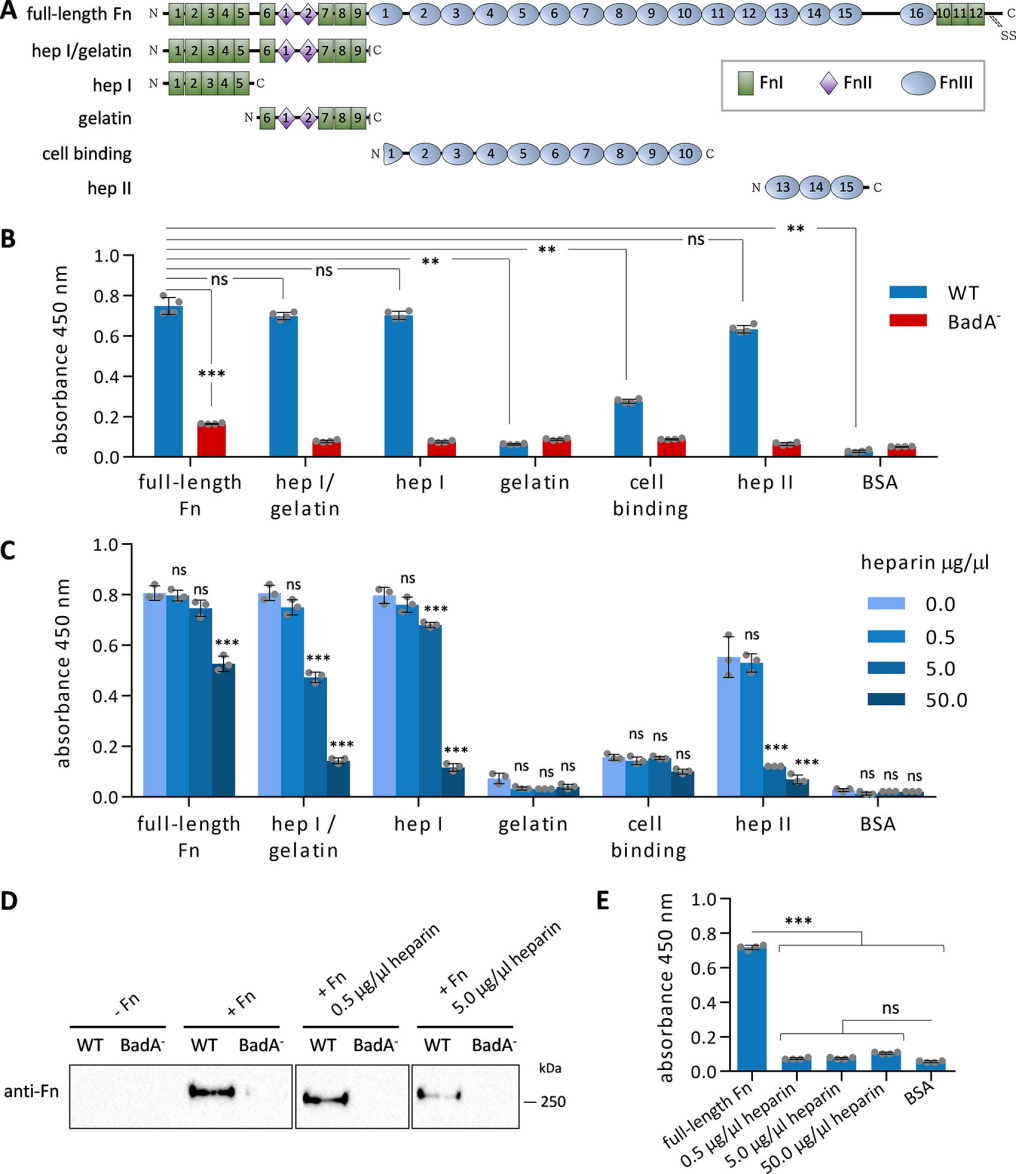
Functional analysis of BadA binding sites in Fn. (A) Schematic representation of monomeric human Fn, composed of 12 type I domains (FnI), 2 type II domains (FnII), and 16 type III domains (FnIII). The distribution of five proteolytic fragments is depicted (detailed mass spectrometry analysis given in Fig. S3). The fragments' affinity to specific extracellular matrix components (based on data from literature) is provided to name the fragments. (B) Characterization of the BadA binding site to immobilized full-length Fn, five Fn proteolytic fragments, and BSA (negative control). Wells were coated with full-length Fn or Fn fragments and incubated with B. henselae WT and BadA^-^. Bacterial binding to Fn was evaluated using anti-B. henselae antibodies. The mean and SD of quadruplicates are depicted. Statistical significance (ns: not significant; ** *P* < 0.0006, *** *P* = 0.0001) was determined using one-way ANOVA with Dunnett’s multiple-comparison test (between all WT samples) and two-tailed unpaired Student's *t* test (between WT and BadA^-^ for full-length Fn). (C, D) Inhibition of B. henselae WT and Fn binding by a competition assay with increased concentrations of heparin using (C) immobilized Fn using an ELISA-based approach (the mean and SD of triplicates are depicted) and (D) Fn in solution using a Western blot approach, without or with Fn (-Fn or +Fn) bound to B. henselae WT and BadA^-^ (protein load 10 μg/lane). Statistical significance was determined using two-way ANOVA with Dunnett’s multiple-comparison test (ns: no significant; *** *P* < 0.0001). (E) B. henselae WT binding to various concentrations of immobilized heparin (controls: Fn and BSA). The mean and SD of quadruplicates are depicted. Statistical significance was determined using one-way ANOVA with Šídák’s multiple-comparison test (ns: no significant; *** *P* < 0.0001).

To identify the BadA-specific interaction sites in Fn, proteolytic Fn-fragments were used in binding assays. The Fn molecule is a heterodimer composed of two splice variants and structurally organized by 12 type I modules (FnI), two type II modules (FnII), and 15–17 type III modules (FnIII) (21). The fragments used in this assay included the heparin I (hep I)/gelatin-binding fragment (FnI 1–9, FnII 1–2), the hep I-binding fragment (FnI 1–5), the gelatin-binding fragment (FnI 6–9, FnII 1–2), the cell-binding fragment (FnIII 2–10), and the heparin II (hep II)-binding fragment (FnIII 13–15), named accordingly to their affinities for other ECM components and cell adhesion molecules (Fig. 3A). The Fn sequence coverage and purity of each fragment were assessed by MS analysis (Fig. S3) revealing that only a few regions of the Fn molecule were not covered by the proteolytic fragments (FnIII 11–12,16, FnI 10–12, see Fig. 3A).

The binding capacity of BadA to each fragment was assessed by bacterial (WT or BadA^-^) adherence to full-length Fn or proteolytic Fn fragments immobilized to microtiter wells via an ELISA approach (Fig. 3B). The data revealed an intense interaction of B. henselae WT to full-length Fn and a strongly reduced interaction of B. henselae BadA^-^. BadA-dependent interactions occurred with the Fn fragments harboring the hep I- or hep II-binding domains without significant differences to the full-length Fn. The cell-binding domain of Fn showed a lesser BadA-dependent interaction with B. henselae, whereas the gelatin-binding region did not show any interaction. The adherence of B. henselae WT to the latter two fragments was strongly reduced compared to full-length Fn.

Competition experiments between bacteria and heparin for binding to the hep I- and hep II-domains in Fn were performed to confirm the functional role of these sites for BadA binding. As a result, heparin reduced bacterial binding to immobilized full-length Fn and the corresponding Fn fragments (i.e., hep I/gelatin, hep I, and hep II fragments) in a dose-dependent manner (Fig. 3C). A slight but not significant reduction of bacterial binding to gelatin and cell-binding domains was also observed at higher heparin concentrations. Similar findings were observed in a suspension-based approach, where bacteria were incubated with heparin, and full-length Fn (Fig. 3D). The possibility that reduced bacterial binding to Fn observed in Fig. 3C and D might be caused by an interfering interaction between B. henselae WT and heparin was excluded as bacteria failed to bind to immobilized heparin (Fig. 3E). Hence, BadA binding to Fn occurs predominantly within the hep I- and hep II-binding domains of Fn.

### Cross-linking mass spectrometry (XL-MS) reveals the interaction sites between BadA and Fn.

**FIG 4:**
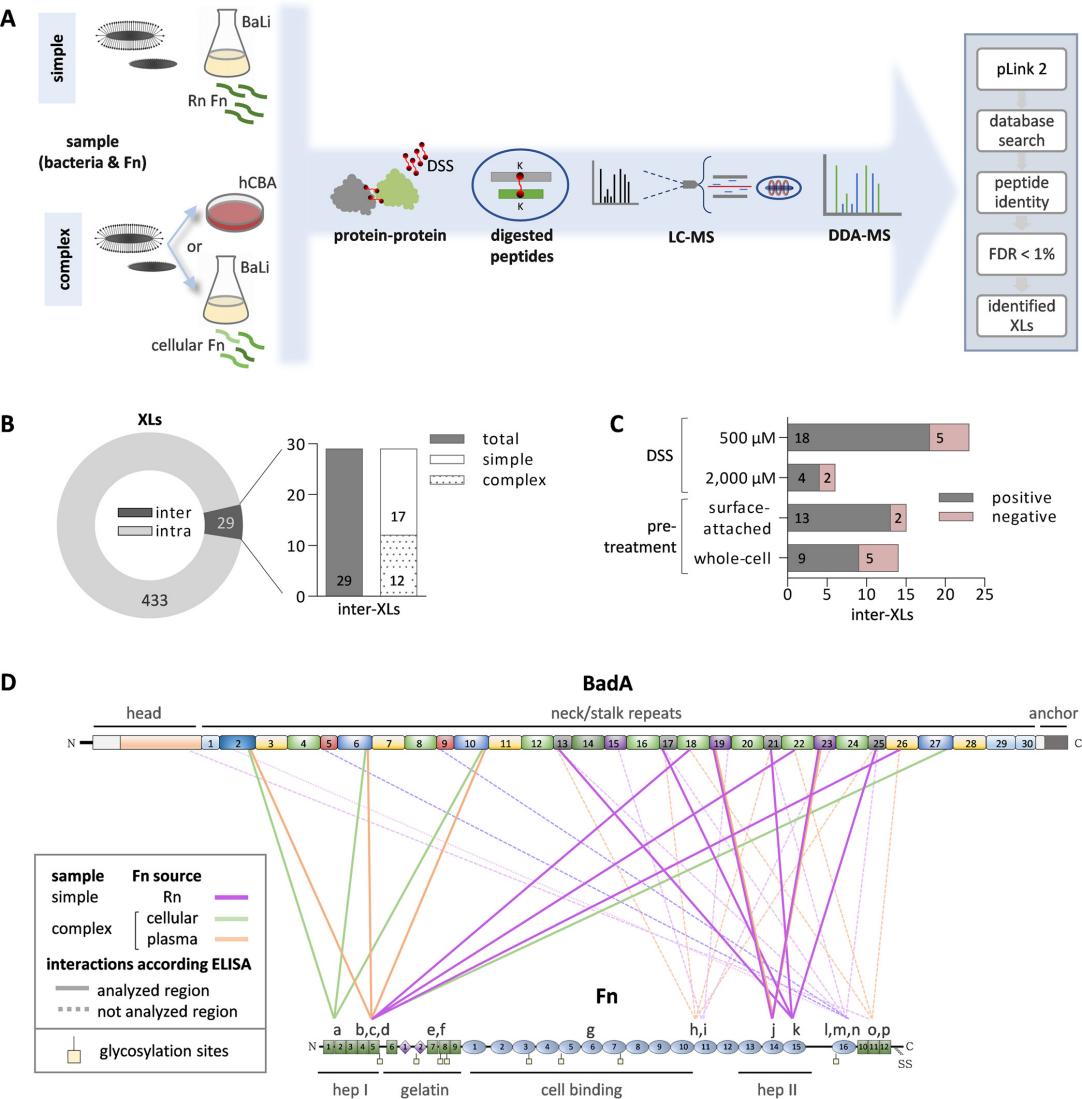
Cross-linking mass spectrometry (XL-MS) reveals the interaction sites between BadA and Fn. (A) Whole-cell B. henselae WT were incubated together with recombinant (Rn) Fn [simple sample], and plasma Fn (hCBA) or cellular Fn [complex samples]. B. henselae BadA^-^ was used as a negative control for identification of false cross-linked peptides pairs. Interacting N-terminal lysine (k) residues were covalently cross-linked using disuccinimidyl suberate (DSS), digested with trypsin, and the peptides were analyzed using DDA-MS. The pLink2 software was used for the identification of cross-links (XLs). The peptide identity was reported with a false discovery rate (FDR) of less than 1%. (B) Total number of BadA and Fn related XLs identified, including intra- (BadA-BadA/Fn-Fn) and inter- (BadA-Fn) XLs. The inter-XLs identified in complex and simple samples are depicted. (C) Inter-XLs identified for each sample type. The effect of different DSS concentrations and sample pretreatment (surface proteins versus whole-cell proteins) was evaluated. Interactions found for positive samples (gray bars, B. henselae WT) and negative-control samples (pink bars, B. henselae BadA^-^) are given. (D) Schematic map of identified cross-linked BadA-Fn interactions. For BadA, the specific BadA regions (head, neck/stalk repeats, and anchor) and similar domains are depicted in matching colors. For Fn, the extension of the proteolytic fragments identified by their interaction with other extracellular matrix components is shown, as well as the glycosylation sites reported in literature (36). The BadA-Fn interacting peptides are indicated (“a”- “p”), the interactions occurring within areas analyzed by ELISA (see Fig. 3 for details) are shown in continuous lines, while interactions occurring in Fn regions for which no proteolytic fragments were available are given in dotted lines. Three XLs within gelatin and cell-binding Fn regions (“e,” “,f” “g”) were not supported by ELISA.

To define the BadA-Fn interaction on a molecular level, B. henselae (whole bacteria) were cross-linked to Fn, followed by data-dependent acquisition MS-based analysis (DDA-MS). Briefly, B. henselae WT or BadA^-^ (the latter as a negative control) were incubated with Fn, followed by extensive washing, and subsequent covalent cross-linking of the N-terminal lysine residues. The cross-linked samples were digested with trypsin and analyzed via MS. Data processing was performed using pLink 2 (Fig. 4A).

As BadA binds both plasma and cellular Fn (Fig. 1C, Fig. S4A), Fn from different sources was used for the cross-linking assays: recombinant (Rn) Fn, cellular Fn, and plasma Fn (adsorbed by B. henselae from hCBA plates as described above); the first of which represents a pure Fn preparation whereas the latter two contain mixtures of different isoforms (as demonstrated for cellular Fn; see Fig. S4B). Furthermore, as whole intact bacteria were used for cross-linking, a combination of approaches was applied to identify as many cross-links (XLs) as possible, including the use of different cross-linker concentrations as well as releasing only the surface-attached B. henselae proteins with cross-linked human proteins to reduce the background noise from intracellular bacterial proteins (see Fig. S5).

The cross-linking analysis revealed 462 XLs corresponding to both intra-protein (BadA-BadA or Fn-Fn) and inter-protein (BadA-Fn) XLs (Fig. 4B). The inter-protein XLs accounted only for 6.3% (*n* = 29) of the total XLs identified, in line with previous observations, where intra-protein cross-links have been demonstrated to account for the majority of observed cross-linked interfaces (22). The identified inter-protein XLs were found in all sample types (Fig. 4B). Most of the identified inter-protein XLs were observed using the lower cross-linker concentration and in the samples where the surface-attached proteins had been released by limited proteolysis (Fig. 4C). All inter-protein XLs identified were included for further analysis, excluding those found in the samples using the BadA^-^ strain accounted for false-positive identifications due to the complex background proteome (Fig. 4C). None of the exact inter-protein XLs found in B. henselae WT samples were identified in B. henselae BadA^-^ samples.

In total, 16 possible BadA-Fn interaction interfaces were identified. Only one XL, corresponding to XL “j,” was identified in more than one sample type, highlighting the complexity of the data in terms of mixed Fn isoforms and other contaminating bacterial and plasma proteins contributing to increased background noise during data analysis (Table 1). The distribution of the identified cross-linked peptides reveals a complex network of interaction at the interface between BadA and Fn, further emphasized by the repetitive domains present in BadA (Fig. 4D). In Fn, the BadA interactions were identified in the hep I- and hep II-binding domains of Fn (Fig. 4D), in line with the above-given ELISA-assays using proteolytic Fn fragments (Fig. 3B). Three XLs were found at gelatin- and cell-binding domains of Fn, which were demonstrated to bind to BadA at a lower avidity (Fig. 3B). Other XLs were identified in regions that were not included in the above described ELISA-based analyses (Fig. 4D, indicated as dotted lines). In BadA, the Fn interactions were mainly distributed along the BadA neck/stalk region and predominantly identified in the repetitive domains (domains: 2/6/10/27, 13/17/21/25, 15/19/23; Fig. 4D). Finally, only one XL (XL “o”) was detected between the BadA head region and the C-terminus of Fn (Fig. S6A), corresponding to a region of the BadA molecule with an available three-dimensional structure. By rendering the BadA head domain (PDB: 3D9X) (23) and the Fn I 11 (PDB: 2EC3) next to each other, it was corroborated that the cross-linked lysine residues are surface exposed, highlighting the feasibility of the suggested interaction interface (Fig. S6B). Notably, the BadA interactions with cellular Fn were observed within the hep I-binding domain; while the interactions with plasma and Rn Fn were detected in hep I- and hep II-binding domains, but mostly within the regions not included in ELISA-assays using proteolytic Fn fragments (Fig. 4D). In conclusion, we identify several possible interaction interfaces between BadA and Fn, which are supported by the complementary ELISA-based assays using proteolytic Fn fragments.

**TABLE 1:** Inter-protein cross-links (XLs) identified between BadA and Fn^*a*^

Sample	Fn source	Fn		BadA		XLs code	Occurrence
		Peptide	Location	Peptide	Location		
Simple	Rn	IGDTWSKKDNR	FnI 5 [hep I]	TVNGEGKEEEK	Stalk [18, 22, 26]	d	1
		IGDQWDKQHDMGHMMR	FnI 7 [gelatin]	VKTVNGEGK	Stalk [14,16,18,20,22,24,26,28]	e	1
		WLPSSSPVTGYRVTTTPKNGPGPTK	FnIII 11	LEKGVSKATQENSK	Stalk [15,19,23]	i	1
		TIKPDVR	FnIII 14 [hep II]	VNNNVTNKFNELTQSITNVTQQVK	Stalk [19,23]	j^*b*^	1
		YEKPGSPPR	FnIII 15 [hep II]	VEDKLTEAVGK	Stalk [13,17,21,25]	k	3
		DQQRHKVR	FnIII 16	VKTVTGEGK	Stalk [2]	l	1
		DQQRHKVR	FnIII 16	GQLDKGLK	Stalk [4-5,8-9]	m	1
		GATYNVIVEALKDQQR	FnIII 16	VEDKLTEAVGK	Stalk [13,17,21,25]	n	1
		WCHDNGVNYKIGEK	FnI 11	DGKKNNVTFDVAR	TRP ring/unpredicted	o	4
Complex	Cellular	PEAEETCFDKYTGNTYR	FnI 2 [hep I]	GASKATQENSK	Stalk [2,6,10]	a	1
		IGDTWSKKDNR	FnI 5 [hep I]	LEKGASK	Stalk [27]	c	1
		IGDQWDKQHDMGHMMR	FnI 7 [gelatin]	LTHVENGDVSEKSK	Stalk [29]	f	1
		IGFKLGVR	FnIII 6 [cell binding]	QMKIVLDDAK	Unpredicted (close to anchor)	g	1
	Plasma (hCBA)	IGDTWSKK	FnI 5 [hep I]	ATQENSKITYLLDGDVSK	Stalk [2,6,10]	b	1
		TEIDKPSQMQVTDVQDNSISVK	FnIII 10 and 11	LTEAVGKVTQQVK	Stalk [13,17,21,25]	h	1
		TIKPDVR	FnIII 14 [hep II]	VNNNVTNKFNELTQSITNVTQQVK	Stalk [19,23]	j^*b*^	1
		IGEKWDR	FnI 11	TVNGEGKEEEK	Stalk [18,22,26]	p	1

a The listed peptides were found in simple and complex samples using pLink 2 program and FDR less than 1.

b XLs found in two different sample types.

## DISCUSSION

Studying the complex interactions of adhesins and host cell receptors can greatly advance our understanding of the molecular mechanisms involved in bacterial adhesion which is the key step of infection. Bacteria use different means to adhere to host cells, and bacterial binding to Fn conceivably is an interesting strategy for colonization due to the ubiquitous distribution of Fn in the pericellular environment. This is corroborated by the large variety of bacterial Fn-binding proteins present in many pathogenic species (21).

BadA represents the principal adhesin of B. henselae. It plays a crucial role in bacterial adhesion to ECM proteins and ECs via an unknown host target, and this was confirmed using BadA-deficient and BadA-complemented B. henselae (7). Expression of BadA is, therefore, the major determinant for B. henselae binding to Fn (Fig. 1, Fig. 2F), despite the description of other FnBPs such as Omp 43, Omp 89, and Pap 31 (24, 25), which are also expressed in the strain used in this study (see Fig. S7). TAA-mediated binding to Fn has also been described for *Yersinia* adhesin A (YadA) from Y. pseudotuberculosis, UpaG from uropathogenic E. coli, and ubiquitous surface protein A (UspA)-1 and -2 from Moraxella catarrhalis (8, 26, 27). The possibility of similar interactions between Fn and other TAAs of pathogenic bacteria needs to be analyzed in greater experimental detail as deciphering similarities in binding motifs in particular TAA’s passenger domains might not be easily predictable due to the reported variable structures within TAAs (10).

Due to the complexity in size and structure of BadA and Fn, we analyzed first the interaction between BadA and Fn using proteolytic Fn fragments to localize regions with strong BadA-binding affinities. Within the Fn regions that are covered by the available fragments, we observed that the hep-binding domains demonstrated the strongest binding to BadA, comparable to the full-length Fn (Fig. 3B), pinpointing their major role for the TAA-mediated adhesion of B. henselae. These Fn regions have been already observed as important interacting sites for adhesins from other pathogenic bacteria (2, 4), in line with the reduced adhesin binding when competition with heparin for Fn binding was evaluated (28). The hep I-binding region in Fn (FnI 2–FnI 5) has been termed as a “canonical binding site” for many FnBPs in Gram-positive (3), and possibly other Gram-negative bacteria (29); while further “non-canonical binding sites” (e.g., hep II-binding domain in Fn) have been also cited as important for other autotransporter adhesins (e.g., ShdA from Salmonella enterica [28]). To our knowledge, BadA is the only adhesin that binds both hep-binding domains in Fn. Overall, the hep-binding domains in Fn seem to be interesting targets to further understand bacterial adhesion to Fn and host cells.

Based on the repetitive nature of BadA, it has been speculated that BadA-mediated adhesion to Fn and ECs might be assisted by the long and modularly arranged neck/stalk region (13). This hypothesis is now confirmed by our XL-MS data, where many regions in the BadA neck/stalk repeats are shown to assist in Fn-binding (Fig. 4D). Notably, the here identified interaction with the domain 27 in the stalk region (Table 1) confirms the earlier demonstrated Fn-binding in a B. henselae mutant expressing a truncated BadA with a short neck/stalk fragment (domains from 27 to 30) (13). Additionally, it was previously speculated that the BadA head might act as a first initiator for bacterial binding via collagen interaction as no Fn-binding was observed for a B. henselae mutant expressing the BadA head fused to a drastically truncated neck/stalk element (12). Here, one interaction between the BadA head and Fn was identified in the XL-MS analysis and theoretically confirmed using available structures (Table 1, Fig. 4D, Fig. S6B). This suggests that although the head of BadA by itself is not strong enough to support detectable bacterial binding to Fn (12), this interaction might act as an accessory binding site for the already strong neck/stalk interactions with Fn.

Our bacterial binding assays demonstrate that the interactions between BadA and the gelatin- or cell-binding domains in Fn are not crucial for BadA binding (Fig. 3B). In accordance, only three XLs (XLs “e,” f,” g”) were detected in these regions (Fig. 4D, Table 1), and two of them (XLs “f,” g”) were identified bound to BadA domains previously described with nonfunctional Fn-binding (13). The importance of other BadA-interaction sites located in Fn-regions that were not covered by proteolytic Fn fragments should be further analyzed in functional binding studies. As described for other adhesins, bacterial binding to host cell surfaces could be accomplished by the avidity of multiple weak binding sites (30) leading to a tighter and effective binding (especially under shear stress). This seems highly “economic” because repetitive elements facilitate recombination events which can modulate the specificity of the adhesin for its biological purpose (10). This was shown for BadA from various B. henselae strains, where variations in the length of the repetitive neck/stalk sequences have been reported but Fn binding was unaffected (31).

Two types of Fn are present in the human body, a globular or soluble (plasma Fn) found in blood, saliva, and other fluids; and a fibril-forming or insoluble (cellular Fn) secreted by fibroblasts and ECs. B. henselae binds both Fn forms (Fig. 1C, Fig. S4A) and according to our XL-MS analysis, BadA interactions to cellular Fn are localized within the FnBPs canonical binding site (hep I-binding domain), while those in plasma Fn and Rn Fn are distributed in multiple sites (i.e., at the end of the hep I-binding domain, hep II-binding domain, and the C-terminus). Due to the different natures of plasma Fn and cellular Fn (32, 33), we speculate that BadA particularly binds regions in cellular Fn (fibrillar structure) that are not exposed in the molecular structure of plasma Fn (e.g., hep I-domain) (33). Also, in cellular Fn, the hep II-domain region has proven to show Fn-Fn interaction in the fibrillar matrix (18) and targeting the hep II-domain for bacterial adhesion might not be feasible in such tight fibrillar conformation. This indicates BadA site-specific interactions with each of the Fn types. B. henselae binding to plasma Fn could, therefore, represent a bacterial strategy to escape the immune response by being masked with plasma Fn, but still retaining the ability to attach to host cells via the pericellular Fn. Such mechanisms may also help to further enhance bacterial adhesion to tissues through the interaction of coated Fn with other host proteins (25).

Protein glycosylation has been described to play a significant role in host-pathogen interaction (34). The glycosylation status of Fn was reported to facilitate the TAA-mediated bacterial adhesion of A. baumannii (35). Our XL-MS data identified interactions in non-glycosylated Fn regions (36), suggesting that many other interactions apart from glycan-protein might occur between Fn and BadA (see Fig. 4D). The role of Fn-glycosylation for BadA binding remained unclear in our approach.

Understanding the underlying molecular mechanisms involved in bacterial adhesion to host tissues is a prerequisite for the development of new therapeutic “anti-adhesive” strategies to prevent pathogen colonization or infection (37). Blocking of the fimbrial adhesin (Fim) by using mannose mimics has already been proposed for preventing uropathogenic E. coli adhesion to bladder cells (38). Thus, anti-ligand strategies seem as interesting alternatives to aid the increasing need for novel antimicrobials which might avoid the selective pressure imposed by bactericidal antibiotics (39).

In conclusion, we mapped the interaction interfaces of a prototypic TAA (BadA of B. henselae) and Fn to mainly occur between the repetitive neck/stalk region of BadA and the hep-binding domains in Fn. Likewise, we demonstrated that the combination of large-scale analysis (XL-MS) approaches to study protein-protein interactions and supportive functional readouts (binding assays) allows for discrimination of crucial interactions involved in bacterial adhesion to the host. The herein described experimental approaches and tools might guide future research for other pathogenic bacteria and our results represent an initial point for future generation of “anti-ligands” to inhibit bacterial binding to host cells as a potential novel therapeutic approach.

## MATERIALS AND METHODS

### Bacterial strains, culture conditions, and reagents.

B. henselae Marseille wild type (WT) and BadA^-^ (7, 40) were cultured for 3 days using *Bartonella* liquid (BaLi) medium (41), supplemented with 2.5% Fn depleted FCS (Sigma-Aldrich, Deisenhofen, Germany) or on human CBA plates (hCBA). NEB 5 alpha competent E. coli (C2987H, NEB, MA, USA) were grown overnight on solid or in liquid lysogeny broth (LB; Becton, Dickinson, Heidelberg, Germany). All bacterial centrifugation steps were performed at 3,800 × *g* for 10 min at 4°C. Bacteria were stored at −80°C in LB supplemented with 20% glycerol (VWR, Darmstadt, Germany).

Fn-depleted FCS was prepared by adding 5 mL of gelatin Sepharose-4B (GE Healthcare, Munich, Germany) to 42 mL of heat-inactivated (56°C, 30 min) FCS and incubated at 4°C in a roller mixer overnight. The gelatin-Sepharose was removed using polypropylene columns (Poly-Prep Chromatography Columns; Bio-Rad, Dreieich, Germany). Filter-sterilized FCS aliquots were stored at −20°C. Fn depletion was confirmed via Western blotting. hCBA was prepared using Columbia agar base (211124, Becton, Dickinson) and defibrinated human blood containing plasma Fn (42).

Fn, proteolytic Fn-fragments, and heparin were purchased from Sigma-Aldrich/Merck Millipore [cellular Fn (F2518), plasma Fn (F2006), recombinant Fn (ECM001), and Fn fragments hep I/gelatin (F0287), hep I (F9911), gelatin (F0162), cell binding (F1904), hep II (F1903), and heparin sodium salt from porcine intestinal mucosa (H4784)]. Antibodies and concentrations are shown in Table S1.

### Mammalian cell culture.

ECs (HUVECs, C-12203, PromoCell, Mannheim, Germany) were cultured as previously described (20) using EC growth media (ECGM, C-22010, PromoCell). For infection experiments, cells were cultured without antibiotics and with Fn-depleted FCS. Lenti-X 293T cells (632180, TaKaRa, Shiga, Japan) were grown using Dulbecco's Modified Eagle Medium (DMEM; Gibco, Karlsruhe, Germany) supplemented with 10% FCS, 1% penicillin-streptomycin, and 1X Glutamax (Thermo Fischer Scientific, Darmstadt, Germany).

### Generation of Fn knockout ECs.

The gRNAs were designed using CRISPOR interface (http://crispor.tefor.net/) (43). LentiCRISPR v2 was a gift from Feng Zhang (Addgene, http://www.addgene.org). Lentiviral CRISPR-Cas9 knockout was carried out as previously described (44). Shortly, the oligos (Table S2) including the overhangs were phosphorylated and annealed using T4 Polynucleotide Kinase (M0201, NEB). Plasmids were digested using FastDigest Esp3I (FD0454, Thermo Fisher) and ligated, including annealed oligos using T7 DNA ligase (M0318, NEB), followed by PlasmidSafe exonuclease treatment (E3110K, Epicentre, WI, USA) according to manufacturer’s guidelines. Plasmids were transformed into E. coli *DH*5 alpha, purified via chloroform-ethanol extraction, and sequenced for confirmation.

Lentivirus was produced by transfection of Lenti-X 293T cells using polyethylenimine (408727, Sigma-Aldrich) with pMD2.G, psPAX2, and the lentiCRISPR v2-gRNAs plasmids (Table S2). pMD2.G and psPAX2 were a gift from Didier Trono (Addgene). Empty lentiCRISPR v2 plasmid was used as a negative control. Viral supernatants were collected after 4 days and filtrated using 0.22 μm filter. Viral presence was confirmed using Lenti-X GoStix Plus (631280, TaKaRa). For transduction of ECs, cells were exposed to the gRNA combination (Fig. 2B) for 24 h followed by puromycin (2 μg/mL; Sigma-Aldrich) selection of positive cells. Cells at passage four were saved in liquid nitrogen for further analysis.

For knockout evaluation, 1.5 × 10^5^ ECs were seeded onto coverslips for immunofluorescence microscopy. For Western blotting, 1 × 10^6^ ECs were seeded onto 6 cm dishes and for RNA quantification, 5 × 10^5^ cells were seeded into six-well plates.

### Relative quantification of *FN1*.

RNA extraction was performed using RNeasy minikit and DNase-treatment (74104 and 79254, Qiagen, Hilden, Germany) following manufacturer’s recommendations. Reverse transcription of extracted RNA was done using LunaScript RT SuperMix kit (E3010, NEB) including a non-RT sample as a control. Amplification of cDNA was performed using Luna Universal qPCR Master Mix (M3003, NEB) and the primers listed in Table S2 on a LightCycler 480 (Roche, Mannheim, Germany) instrument. The mRNA relative quantification of *FN1* gene was normalized using *beta-actin* as a housekeeping gene. The Ct values from each gene were obtained to calculate the average normalized fold expression ($2^{-\Delta \Delta \text{Ct}}$). The ΔCt value was calculated for the empty vector (control) and knockout treatments ΔCt=Ct(FN1) − Ct(beta − actin). The ΔΔCt was calculated according to ΔΔCt=ΔCt(test) − ΔCt(control) and converted to normalized fold expression as previously reported (45).

### Bacterial binding to Fn in solution.

Bacteria were grown in BaLi medium and washed twice with Dulbecco's phosphate-buffered saline (DPBS; Gibco). Bacterial binding to purified plasma, cellular, and Rn Fn was performed using 5 × 10^8^ bacteria (optical density [OD]; 1.0 OD = 5 × 108 cells/mL) and 7.5 μg Fn in DPBS. For cross-linking analysis, samples were incubated for 30 min at 37°C while gently shaking. For competition binding experiments, 0.5 or 5 μg/μL heparin were added and incubated at 37°C for 2 h while shaking. After each incubation step, cells were centrifuged and washed three times with DPBS.

### Bacterial binding to immobilized Fn.

Bacterial adhesion to immobilized Fn was evaluated using ELISA. Full-length and Fn fragments were coated onto Nunc Maxisorp flat-bottom 96-wells (468667, Thermo Scientific) using 1 μg, unless mentioned otherwise. Plates were blocked with 2% wt/vol bovine serum albumin in DPBS. Bacteria grown in BaLi medium (1.5 × 10^8^ bacteria, based on OD, see above) were added and incubated for 2 h at 37°C. For competition experiments with immobilized Fn, different concentrations of heparin and bacteria in DPBS were used in Fn-coated wells. For bacterial binding assays to heparin, 0.5, 5, or 50 μg/μL of heparin was used for coating. Interaction of BadA with Fn or heparin was examined by whole-cell ELISA using rabbit anti-B. henselae IgG antibodies followed by HRP conjugated anti-rabbit IgG antibodies in blocking buffer. After each step, three washes were performed using 0.05% vol/vol Tween 20 in DPBS. The assay was developed using TMB solution (T4444, Sigma-Aldrich) and absorbance was spectrophotometrically measured at 450 nm.

### Bacterial adhesion assays.

ECs were treated as previously described (20) with some modifications. Briefly, for immunofluorescence, 1.5 × 10^5^ ECs were seeded onto collagenised coverslips in 24-well plates, grown overnight without antibiotics, and infected with a multiplicity of infection (MOI) of 200 for 60 min. The number of viable bacteria in stock vials was quantified from culturing logarithmic dilutions of bacteria on CBA and subsequent counting of CFU (CFU). After infection, three washes with ECGM were performed to remove unbound bacteria. For absolute quantification of adherent bacteria via qPCR, 5 × 10^5^ ECs were seeded into six-well plates and infected as described above.

### Immunofluorescence microscopy.

ECs were fixed using 3.75% paraformaldehyde (PFA) for 10 min at 4°C, permeabilized with 0.1% Triton X-100 for 15 min and blocked with 1% BSA for 1 h (both dissolved in DPBS; both from Sigma-Aldrich). Primary and secondary IgG-antibodies (rabbit anti-B. henselae, mouse anti-cellular fibronectin, Cy5 conjugated anti-mouse, Alexa 488 conjugated anti-mouse, Alexa 488 conjugated anti-rabbit, Alexa 488 conjugated rabbit anti-laminin, Alexa 647 conjugated rabbit anti-collagen V) were incubated at room temperature for 1 h. Actin cytoskeleton was stained with TRITC phalloidin. Bacterial and mammalian DNA was stained with DAPI for 10 min. Three washes with DPBS were performed between each step. Coverslips were mounted using fluorescence mounting medium (S3023, Dako, Hamburg, Germany). Slides were examined using a Zeiss Axio Imager 2 microscope (Zeiss, Oberkochen, Germany) equipped with a Spot RT3 microscope camera (Diagnostic Instruments Inc, MI, USA) and operated by VisiView V.2.0.5 (Visitron Systems, Puchheim, Germany).

### qPCR quantification of adherent bacteria.

Bacteria adherent to ECs were quantified by the numbers of gene copy equivalents. Infected cells were scraped off, pelleted at 20,000 × *g* for 3 min, and washed once with DPBS. For DNA extraction, the cell pellet was resuspended in alkaline lysis buffer (25 mM NaOH, 0.2 mM EDTA) and boiled at 95°C for 30 min. The sample was cooled at 4°C and neutralization buffer (40 mM Tris HCl, pH 7.8) was added. The sample was centrifuged at 10,000 × *g* for 10 min, and the supernatant was transferred to a new tube.

The isolated genomic DNA was used to amplify species-specific genes for the determination of bacterial and ECs gene copy numbers as previously described (46). Shortly, the number of adherent B. henselae was quantified by using a *glyA* fragment (serine hydroxymethyltransferase, 120 bp), and the number of ECs was identified using a *hmbs* fragment (hydroxymethylbilane synthase, 207 bp). For each gene, a standard control was produced by ligation of the PCR product of *glyA* or *hmbs* genes into a pCR 2.1-TOPO vector (Table S2) following the manufacturer´s recommendations. DNA was amplified using Luna Universal qPCR Master Mix and 0.25 μM forward and reverse primers (see Table S2). The copy numbers of the resulting standard plasmids were calculated following the formula: $\text{copy}\;\text{numbers}=\frac{N_{A}\times n}{M}$; where $N_{A}$ is the Avogadro constant number $\left(6.02\times 10^{23}mol^{-1}\right)$, *n* is the plasmid concentration, and *M* is the plasmid molecular mass. For each qPCR, an internal standard with the calculated gene copy numbers was included. For absolute quantification of adherent bacteria per EC, the following formula was used: $\text{binding}\;\text{ratio}=\frac{\text{glyA}\;\text{gene}\;\text{equivalent}}{0.5\;\text{hmbs}\;\text{gene}\;\text{equivalent}}$.

### Immunoelectron microscopy.

Bacteria bound to Fn in solution were used for immunoelectron microscopy. After centrifugation, cells were fixed using 3% PFA and 0.02% glutaraldehyde for 3 h at 4°C. Fixed cells were washed once with DPBS and kept at 4°C for 18 h. After centrifugation, the sediment was embedded in 4% agarose (Sigma-Aldrich) at 37°C and then cooled on ice. Agarose blocks were embedded in Lowicryl (Polysciences Ltd., Hirschberg an der Bergstraße, Germany). Ultrathin sections (50 nm) were mounted on Formvar-coated nickel grids and incubated with rabbit anti-fibronectin, followed by 6 nm gold-conjugated goat anti-rabbit IgG. In control samples, the primary antibody was omitted. Grids were examined using a transmission electron microscope (Zeiss LIBRA 120; Carl Zeiss, Oberkochen, Germany).

### Western blotting.

Bacterial proteins were extracted using Laemmli sample buffer (S3401, Sigma-Aldrich) and samples were heat-denatured at 95°C for 5 min. ECs were collected using 200 μL of protein sample buffer (7 M urea, 1% SDS, 10% glycerol, 10 mM Tris-HCl pH 6.8, 5 mM DTT, all Sigma-Aldrich) containing cOmplete Protease Inhibitor Cocktail (04693124001, Roche) and proteins were prepared in Laemmli sample buffer. Proteins were separated by SDS-PAGE, transferred to nitrocellulose membranes, and tested using primary (overnight, 4°C) and HRP-conjugated secondary (room temperature, 1 h) antibodies. For detection, blots were developed using SuperSignal West Pico PLUS Chemiluminescent Substrate (34577, Thermo Scientific) and a ChemiDOC XRS+ system equipped with ImageLab V6.0.1. software (Bio-Rad). Primary (rabbit anti-BadA, mouse anti-fibronectin, rabbit anti-GAPDH) and secondary (HRP conjugated anti-rabbit IgG or anti-mouse IgG) antibodies were used for protein detection.

### Protein cross-linking sample preparation.

Cross-linking was performed as previously described (17) with some modifications. Bacteria bound to Fn in solution were resuspended in DPBS and heavy/light DSS (001S, DSS-H12/D12, Creative Molecules Inc.) was added to a final concentration of either 500 or 2,000 μM and subsequently incubated for 60 min at 37°C with gently shaking. The cross-linking reaction was quenched with 50 mM ammonium bicarbonate (Sigma-Aldrich) for 15 min at 37°C. Samples were split into surface-attached proteins and whole-cell proteins. Surface-attached proteins were recovered by limited proteolysis using 2 μg of sequencing grade trypsin (V511A, Promega, Lyon, France) for 2 h at 37°C while shaking. Cell debris was removed by centrifugation to recover the supernatant. Surface-attached and whole-cell proteins were heat-inactivated at 85°C for 5 min and used for MS sample preparation.

### MS sample preparation.

All samples for MS analysis were prepared by denaturing the proteins using 8 M urea/100 mM ammonium bicarbonate solution. Cysteine bonds were reduced using 5 mM Tris (2-carboxyethyl) phosphine hydrochloride (646547, Sigma-Aldrich) for 60 min at 37°C and alkylated using 10 mM 2-iodoacetamide (Sigma-Aldrich) for 30 min at 22°C. For digestion of cross-linked samples, 1 μg lysyl endopeptidase (125-05061, Wako Chemicals, Neuss, Germany) was added, and samples were incubated for 2 h at 37°C. All samples were diluted with 100 mM ammonium bicarbonate to a final urea concentration of 1.5 M, and 1 μg sequencing grade trypsin was added for 18 h at 37°C. The digested samples were acidified with 10% formic acid (Sigma-Aldrich) to a pH of 3.0. Peptides were purified and desalted using SOLAμ reverse phase extraction plates (60509-001, Thermo Scientific) following the manufacturer’s recommendations. Dried peptides were reconstituted in a solution containing 2% acetonitrile (Sigma-Aldrich) and 0.1% formic acid before MS analysis.

### Liquid chromatography MS.

All peptides were analyzed on a Q Exactive HFX connected to an EASY-nLC 1,200 (both Thermo Scientific). The peptides were separated on a Thermo EASY-Spray column (Thermo Scientific 50 cm column, column temperature 45°C) operated at a maximum pressure of 800 bar. A linear gradient of 4% to 45% acetonitrile in aqueous 0.1% formic acid was run for 50 min for both data-dependent acquisition (DDA) and data-independent acquisition (DIA).

For DDA analysis, one 238 full MS scan (resolution 60,000 for a mass range of 350-1,600 *m/z*) was followed by MS/MS scans (resolution 15,000) of the 15 most abundant ion signals. The precursor ions with 2 *m/z* isolation width were isolated and fragmented using higher-energy collisional-induced dissociation (HCD) at a normalized collision energy of 30. The automatic gain control (AGC) was set as 3e6 for full MS scan and 1e5 for MS/MS and the dynamic exclusion was set to 10 s. For DIA, a full MS scan (resolution 60,000 for a mass range of 390-1,210 *m/z*) was followed by 32 MS/MS full fragmentation scans (resolution 30,000) using an isolation window of 26 *m/z* (including 0.5 *m/z* overlap between the previous and next window). The precursor ions within each isolation window were fragmented using HCD at a normalized collision energy of 30. The AGC was set to 3e6 for MS and 1e6 for MS/MS.

Quantitative MS data analyses were stored and managed using openBIS (47). All MS raw data were converted to gzipped and Numpressed mzML using the tool MSconvert from the ProteoWizard, v3.0.5930 suite (48, 49). DDA acquired spectra were analyzed using the search engine X! Tandem (2013.06.15.1-LabKey, Insilicos, ISB), OMSSA (version 2.1.8), and COMET (version 2014.02 rev.2) (50–52) against an in-house compiled database containing the Homo sapiens reviewed and B. henselae unreviewed proteomes (UniProt proteome IDs UP000005640 and UP000000421, respectively), yielding a total of 21,846 protein entries and an equal amount of reverse decoy sequences. Fully tryptic digestion was used allowing two missed cleavages. Carbamidomethylation (C) was set to static and oxidation (M) to variable modifications, respectively. Mass tolerance for precursor ions and fragment ions was set to 0.2 Da and 0.02 Da, respectively. Identified peptides were processed and analyzed through the Trans-Proteomic Pipeline (TPP v4.7 POLAR VORTEX rev 0, Build 201403121010) using PeptideProphet (53). The false discovery rate (FDR) was estimated with Mayu (version 1.07) and peptide spectrum matches (PSMs) were filtered with protein FDR set to 1% resulting in a peptide FDR < 1%.

The DIA data were processed using the OpenSWATH pipeline (version 2.0.1 revision: c23217e) (54). Spectral libraries from the above DDA data set were created in openBIS using SpectraST (version 5.0, TPP v4.8.0 PHILAE, build 201506301157-exported [Ubuntu-x86_64]) in TPP (55). The retention time (RT) extraction window was ±300 s, and *m/z* extraction was set at 0.05 Da tolerance. RT was calibrated using iRT peptides. Peptide precursors were identified by OpenSWATH (2.0.1) and PyProphet (2.0.1) was used to control the FDR of 1% at peptide precursor and protein level. TRIC was enabled (56) but realigned and requantified values were subsequently removed. The resulting DIA data sets were analyzed using Jupyter Notebooks (version 3.1.1).

The peptide sequence coverage of the proteolytic Fn fragments and the Fn isoform distribution were analyzed from DDA acquired spectra with PEAKS (version X) against the UniProt Homo sapiens reviewed proteome, as above. Tryptic digestion was used allowing for two missed cleavages. Carbamidomethylation (C) was set to static and oxidation (M), deamination (N and Q) to variable modifications. Mass tolerance for precursor ions was set to 5 ppm and 0.02 Da for fragment ions. Maximum of posttranslational modifications (PTM) per peptide was 2. The search results were filtered using 1% FDR and 2 unique peptides.

### Cross-linking data analysis.

All spectra from cross-linked samples were analyzed using pLink 2 (version 2.3.9). pLink2 was run using default settings for conventional DSS-H12/D12 cross-linking, with trypsin as the protease and an allowance of up to three missed cleavages. Peptides with a mass range of 35-8,000 *m/z* were selected, and the fragment and precursor tolerance were set to 10 and 20 ppm, respectively. The target protein database contained BadA (GenBank: MK993576.1) and human Fn (UniProt: P02751-1). Fn isoform 1 was used as input for all Fn sample types used in this research. Rn Fn was produced in HEK 293 cells using this isoform 1 (according to the manufacturer´s specifications). In the case of cellular Fn, although the most prominent isoform was number 14 (according to DDA-MS analysis, Fig. S8), a pLink 2 analysis for cellular Fn samples, including as a database the BadA and isoform 1 or 14 sequences produced the same cross-linked peptides (data not shown). The results were processed using a filter tolerance of 20 ppm and an FDR of 1%. All cross-linked peptides were filtered based on an E-value <1. The Fig. S6B was generated using PyMOL V2.5 (The PyMOL Molecular Graphics System, Schrödinger, LLC.)

### Data analysis and statistics.

All experiments were performed at least two times, the number of replicates is depicted in each figure. The XL-MS analysis was performed once in triplicates. For immunofluorescence microscopy, representative pictures from at least 25 high-power fields are depicted. All statistical analyses were performed using Prism V6 (GraphPad Software, San Diego, CA, USA) assuming data parametric distribution. A value of *P* < 0.01 was considered statistically significant. The specific test for each analysis and *P*-value are described in the corresponding figure legend.

### Data availability.

The mass spectrometry data presented in this study have been deposited in the ProteomeXchange consortium via the MassIVE partner repository (https://massive.ucsd.edu/) with the identifier PXD032840.
