# Supplementary material for: Interaction of Bartonella henselae with Fibronectin Represents the Molecular Basis for Adhesion to Host Cells
Source: Microbiol Spectr. 2022 Apr 18;10(3):e00598-22. doi: 10.1128/spectrum.00598-22 (PMC9241615; doi:10.1128/spectrum.00598-22)
Supplement: SUPPLEMENTAL FILE 1 — Supplemental material. Download spectrum.00598-22-s001.pdf, PDF file, 3.1 MB [file spectrum.00598-22-s001.pdf]

Interaction of *Bartonella henselae* with fibronectin represents the molecular basis for adhesion to host cells

Diana J. Vaca, Arno Thibau, Matthias S. Leisegang,
Johan Malmström, Dirk Linke, Johannes A. Eble, Wibke Ballhorn,
Martin Schaller, Lotta Happonen, Volkhard A. J. Kempf

Table of contents

Supplementary Figures ... 2

Figure S1. Immunolabeling of Fn bound to *B. henselae* or expressed by endothelial cells (ECs). ... 2

Figure S2. Immunofluorescence microscopy of CRISPR Cas-mediated Fn knockout human endothelial cells (ECs). ... 3

Figure S3. Analysis of purity and sequence coverage of full-length Fn and proteolytic fragments of Fn. ... 4

Figure S4. *B. henselae* binding to cellular Fn and cellular Fn isoforms. ... 8

Figure S5. Identification of BadA and Fn abundance in *B. henselae* WT and BadA$^-$ samples grown on hCBA plates. ... 9

Figure S6. Analysis of crosslink "o" BadA head and C-terminus of Fn. ... 10

Figure S7. Abundance of outer membrane Fn binding proteins in *B. henselae* WT and BadA$^-$. ... 11

Figure S8. Analysis of most prominent isoform in cellular Fn. ... 12

Supplementary Tables ... 13

Table S1. Antibodies (IgG) and staining chemicals used in this study. ... 13

Table S2. Oligonucleotides and plasmids used in this study. ... 14

Supplementary References ... 15

Supplementary Figures

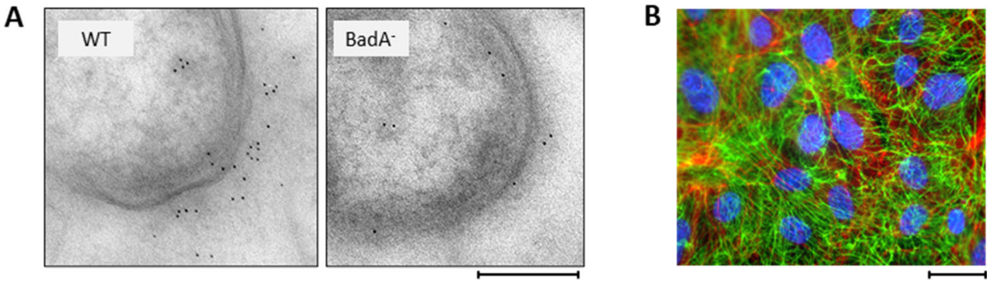

Figure S1. Immunolabeling of Fn bound to *B. henselae* or expressed by endothelial cells (ECs).
(A) Immunoelectron microscopy of Fn (black dots) bound to WT (left) and BadA^{-} (right) bacteria. Fn was detected with anti-Fn primary and gold-labeled secondary antibodies (scale bar: 200 nm).
(B) Immunofluorescence microscopy of ECs demonstrating the presence of Fn covering ECs in its extracellular environment (Fn: green; nuclei: blue; beta-actin: red. Scale bar: 30 µm).

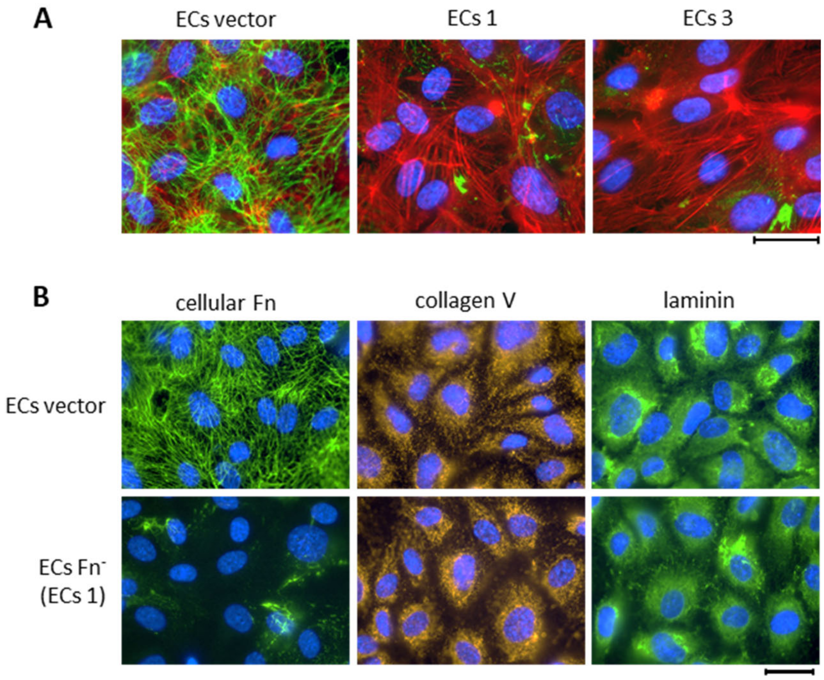

Figure S2. Immunofluorescence microscopy of CRISPR Cas-mediated Fn knockout human endothelial cells (ECs). (A) CRISPR Cas-mediated Fn knockout ECs. ECs vector contained an empty vector (for WT phenotype), ECs 1 and ECs 3 demonstrated efficient Fn removal (Fn: green; nuclei: blue; beta-actin: red. Scale bar: 30 µm). **(B)** Cellular Fn, collagen V, and laminin were stained in ECs vector and ECs Fn^- (ECs 1) cells to confirm Fn removal and to exclude an impact on collagen and laminin arrangement in the pericellular environment (Fn or laminin: green; collagen V: orange; nuclei: blue. Scale bar: 30 µm).

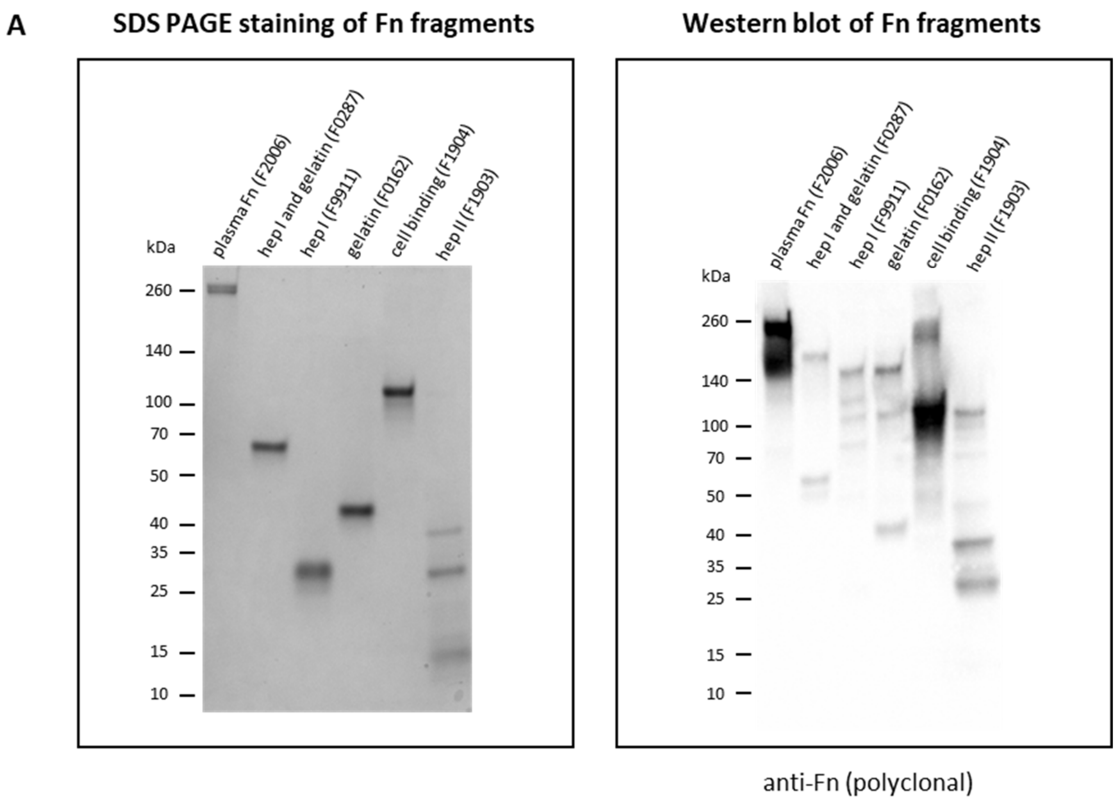

Figure S3. Analysis of purity and sequence coverage of full-length Fn and proteolytic fragments of Fn. (A) Analysis of Fn purity of Fn full-length and proteolytic Fn fragments via SDS PAGE (left) and Western blotting (right) using anti-Fn polyclonal antibodies.

B

hep I and gelatin-binding fragment (F0287)

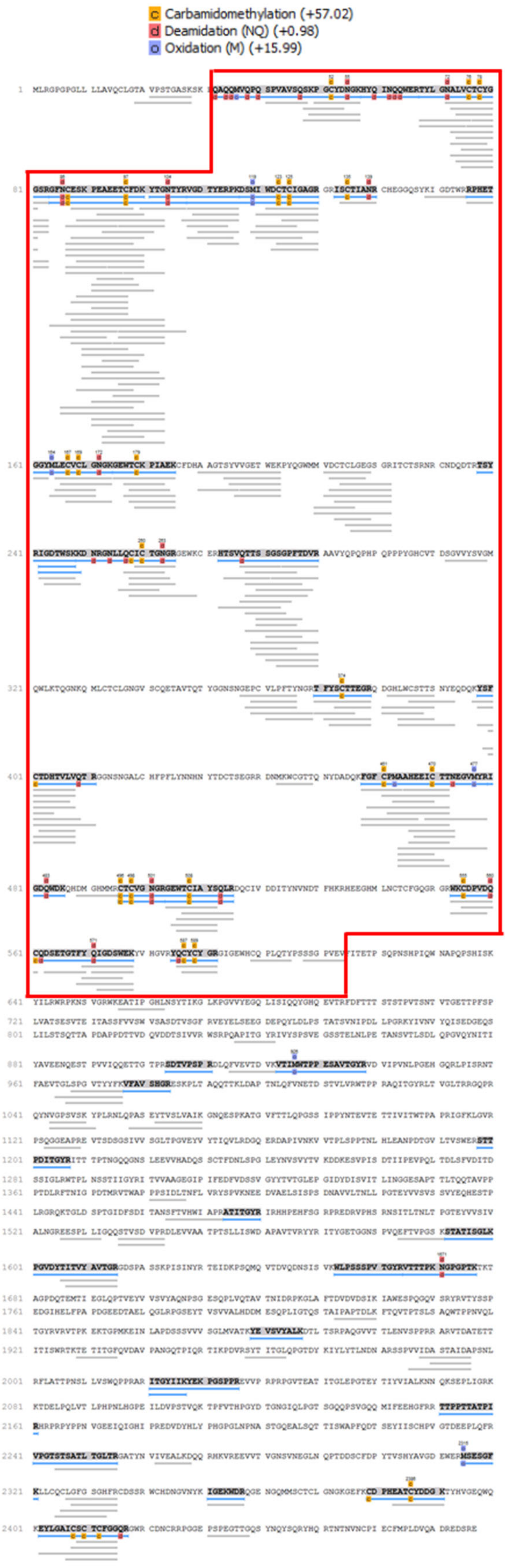

sequence: p32-p290; 66 kDa

hep I-binding fragment (F9911)

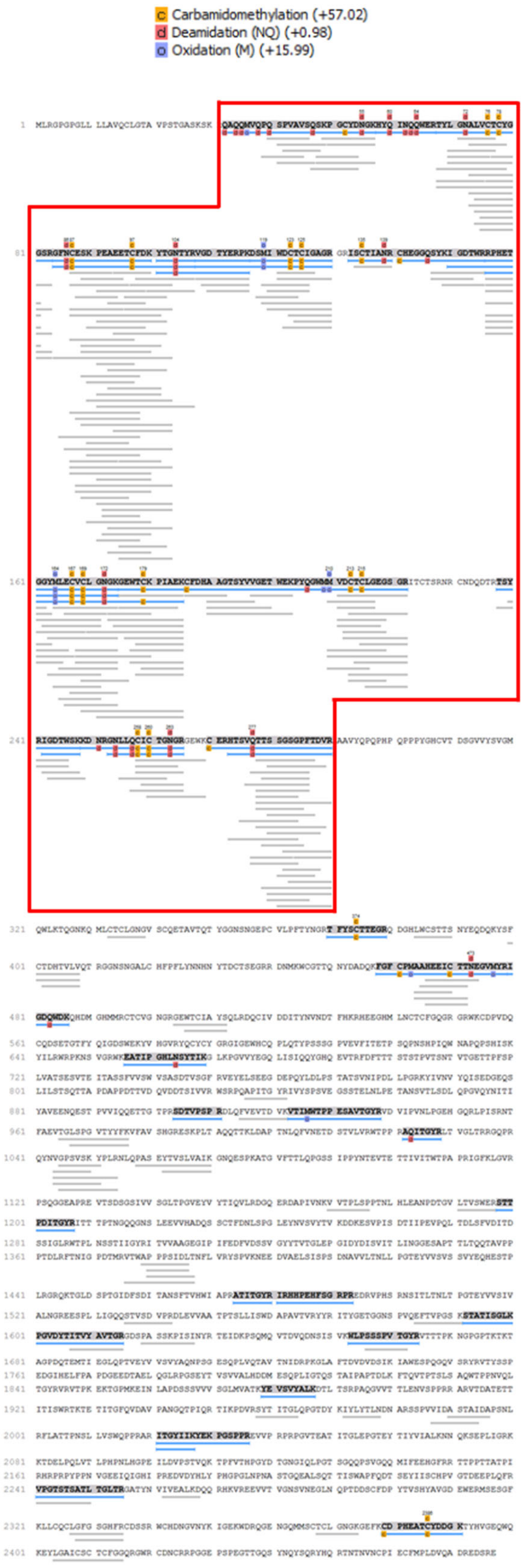

sequence: p32-p290; 29 kDa

B

gelatin-binding fragment (F0162)

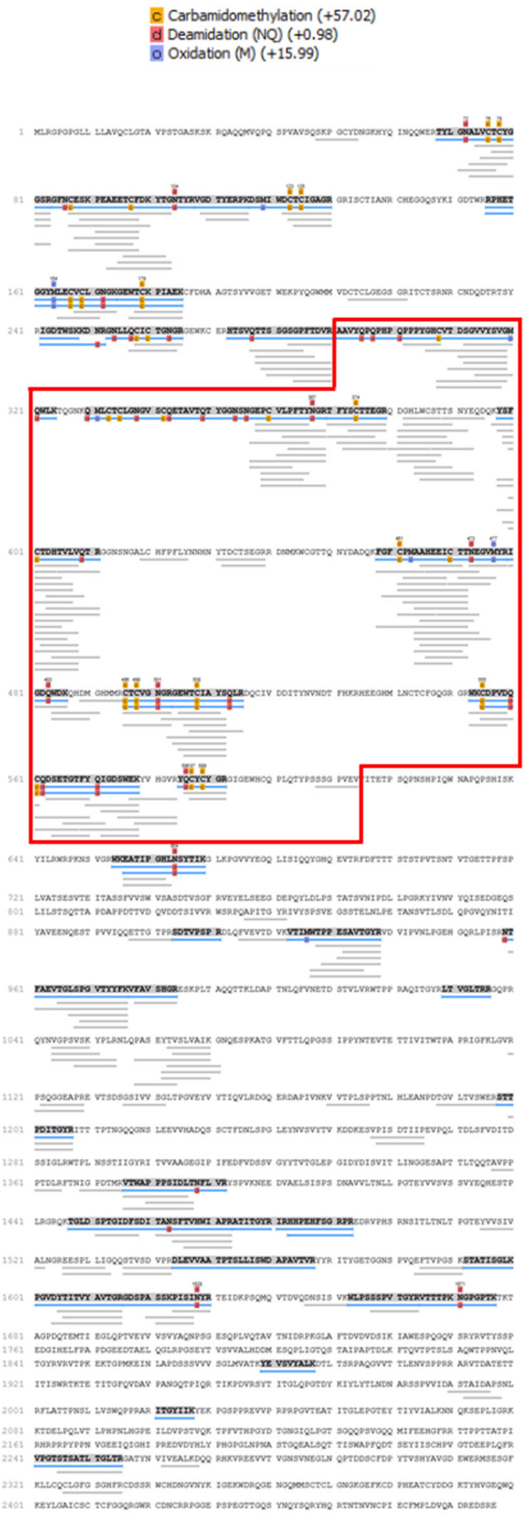

sequence: p291 – p614; 37 kDa

cell-binding fragment (F1904)

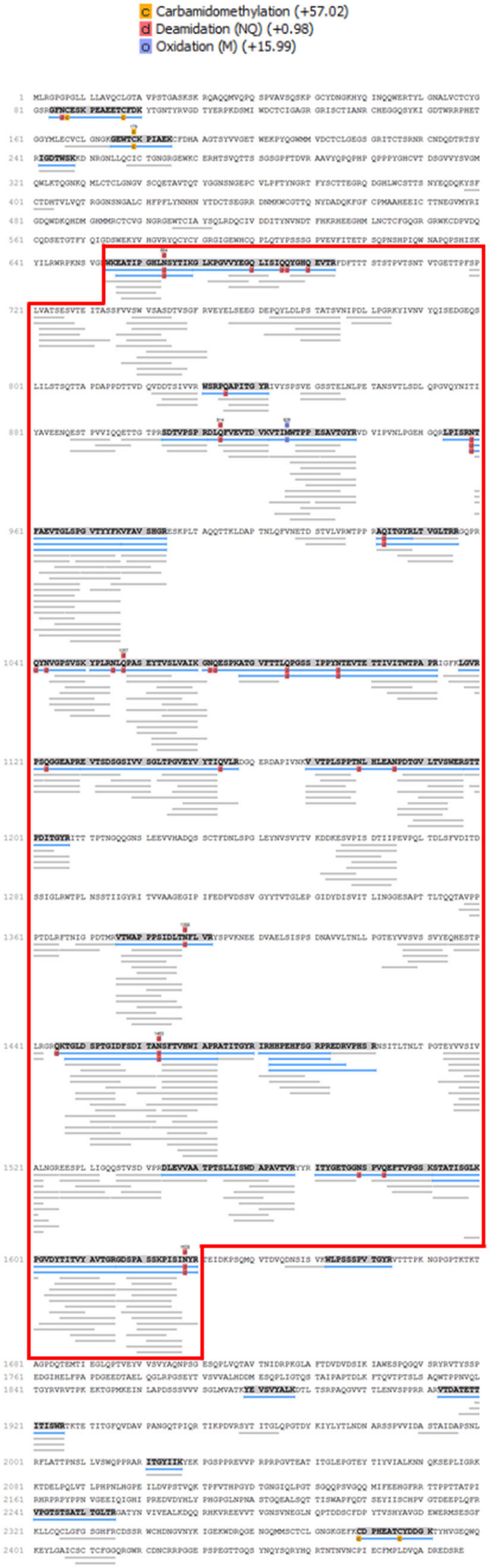

sequence: p654 – p1540; 96 kDa

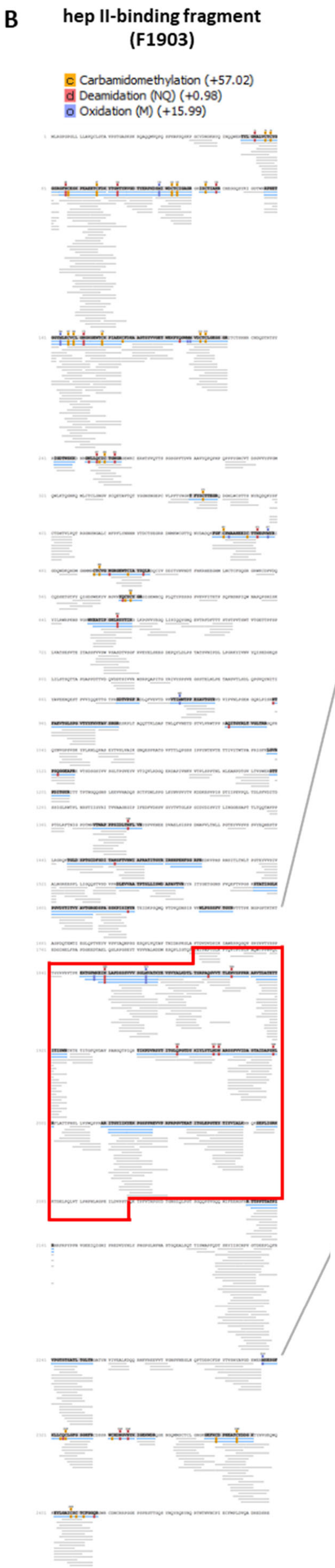

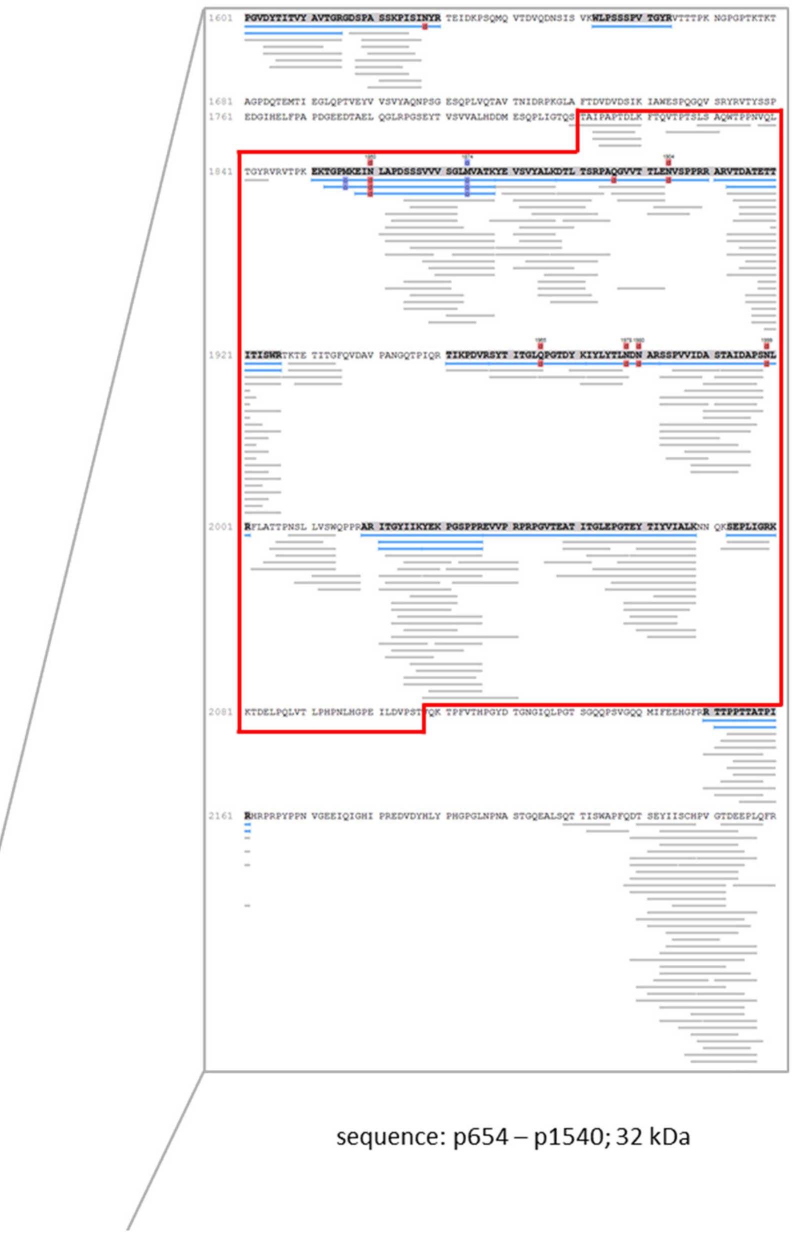

Figure S3. Analysis of purity and sequence coverage of full-length Fn and proteolytic fragments of Fn. (B) DDA-MS analysis of proteolytic Fn fragments. The sequence coverage of each fragment is depicted below the sequence as blue bars (grey bars are based on *de novo* identification) with the sites of post-translational modifications indicated in yellow (carbamidomethylation cysteine [C]), red (deamidation asparagine [N], or glutamine[Q)], and blue (oxidation methionine [M]) boxes. The suggested sequenced coverage is indicated in each case with a red outline and is based on both the DDA-MS analysis and the manufacturer product information. Note that the hep II-binding fragment (F1903) is not completely pure and contains also other parts of the Fn molecule. The other identified peptides for this fragment included hits from the hep I-binding domain and the C-terminal part of the Fn sequence, which might be related to the purification procedure for this fragment (chymotryptic digestion of human plasma Fn and purified with heparin-sepharose column chromatography according to manufacturer´s specification).

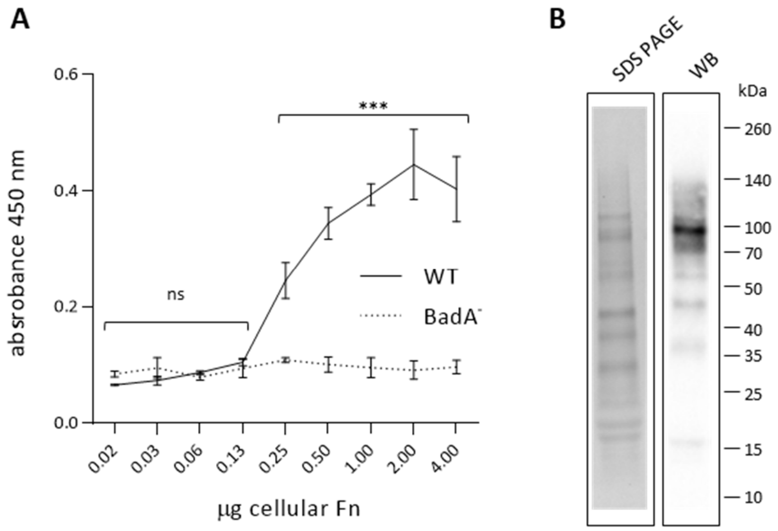

Figure S4. ***B. henselae* binding to cellular Fn and cellular Fn isoforms. (A)** Binding of *B. henselae* WT and BadA$^-$ (1.5x10^8 cells) to increasing coating concentrations of cellular Fn. Bacteria were detected using anti-*B. henselae* antibodies in an ELISA format. The mean and SD of triplicates are depicted. Statistical significance was determined using a two-way ANOVA with Šídák's multiple comparisons test between WT and BadA$^-$ (ns: not significant, *** $p<0.0001$). **(B)** Analysis of cellular Fn (note the presence of different Fn lengths) via SDS PAGE (left) and (WB) Western blotting using anti-Fn polyclonal antibodies (right).

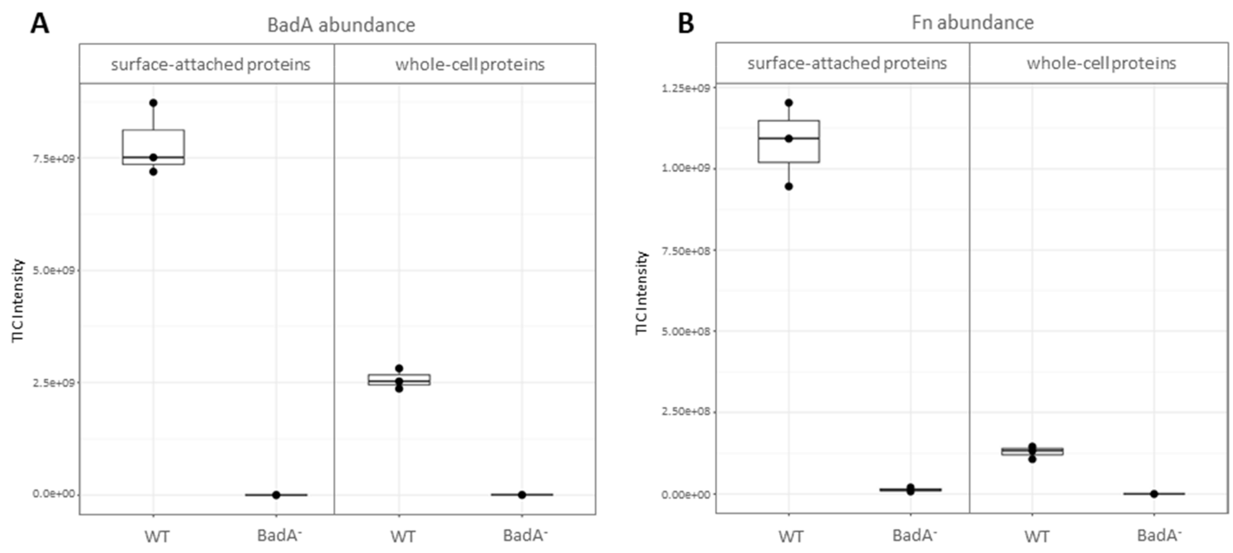

Figure S5. Identification of BadA and Fn abundance in *B. henselae* WT and BadA⁻ samples grown on hCBA plates. DIA-MS-based quantitation of **(A)** BadA and **(B)** Fn abundance in the different sample types used in the experimental crosslinking setup (see Material and Methods).

A

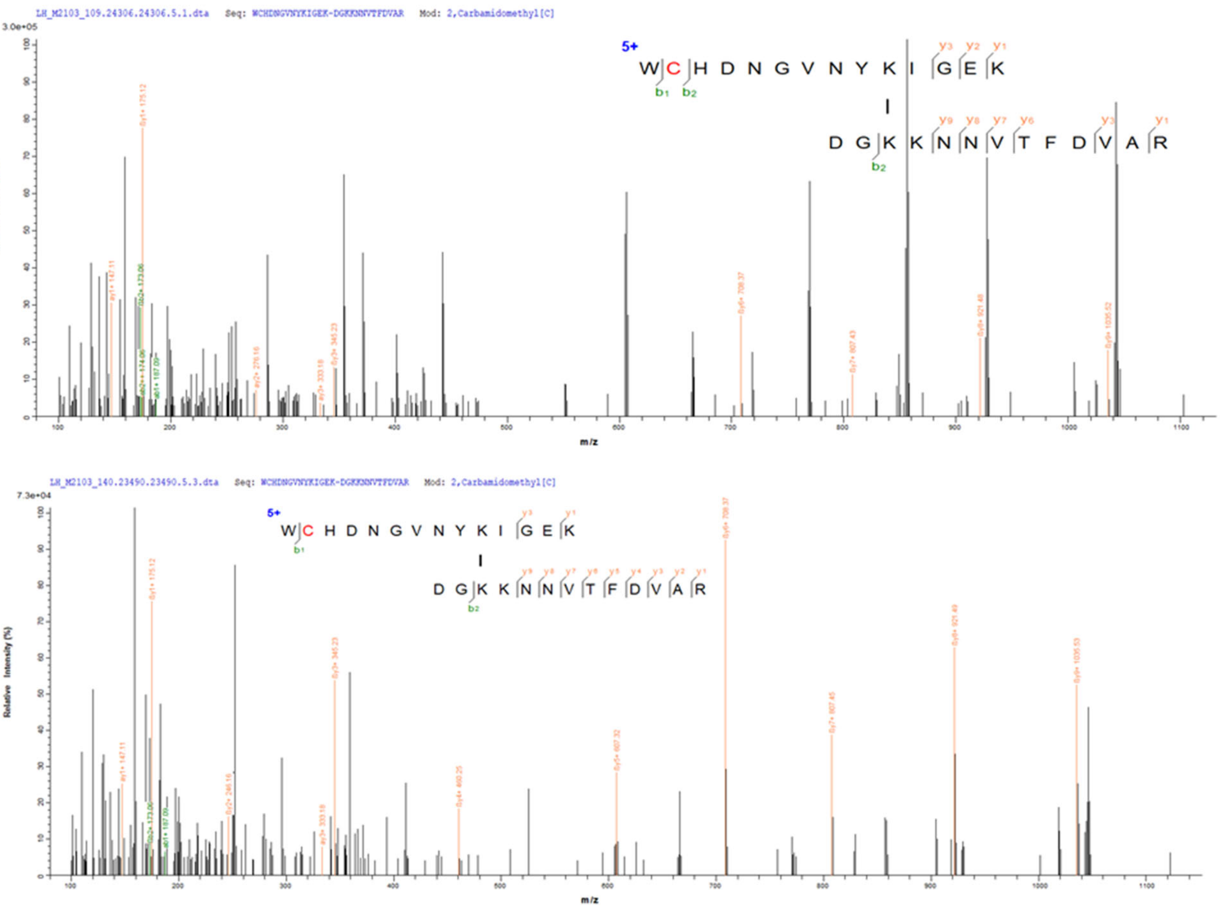

B

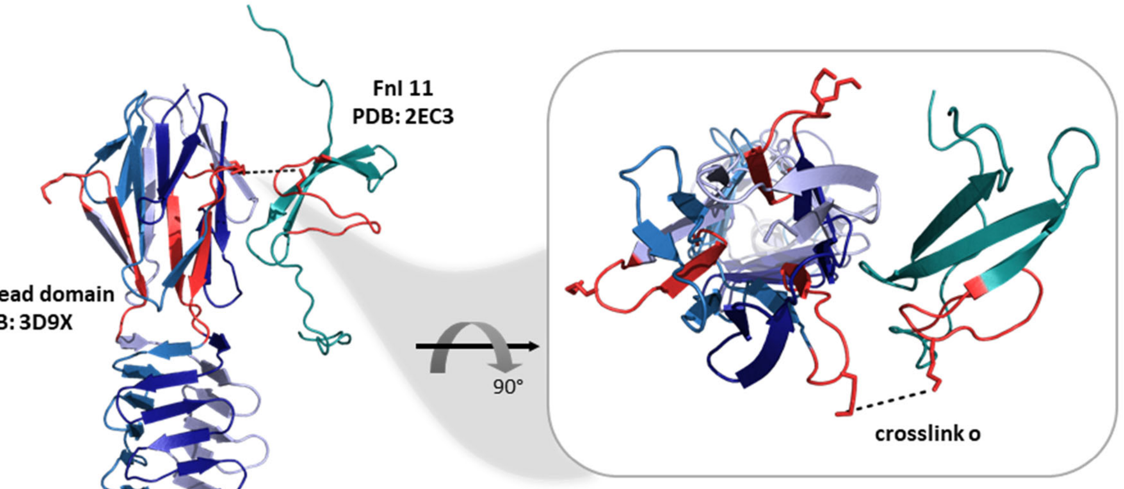

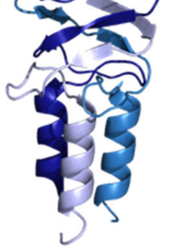

Figure S6. Analysis of crosslink "o" BadA head and C-terminus of Fn. **(A)** Sample spectra obtained from pLink for the crosslink "o". The upper panel displays the name of the file, followed by the sequence (Seq) of crosslinked peptides and modification (Mod) carbamidomethylation of cysteine [C]. Identified crosslinked peptides including matched b/y ions are shown in the annotated spectrum. **(B)** The BadA head domain crystal structure (PDB: 3D9X) (1) rendered next to the FnI 11 NMR structure (PDB: 2EC3). The three different BadA monomers are shown in shades of blue, and the FnI 11 domain in dark cyan. The peptides identified as crosslinked are indicated in red (crosslink "o" in **Table**), and the identified crosslink between one of the BadA monomers and the Fn fragment is indicated as a black dotted line drawn between the lysine (K) residues. The figure was generated in pyMOL.

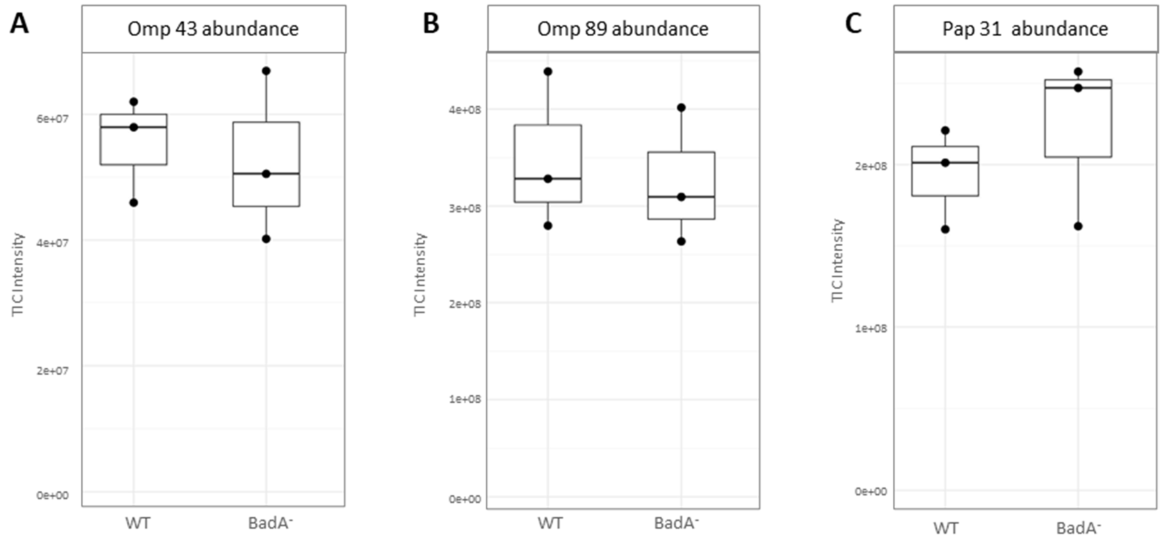

Figure S7. Abundance of outer membrane Fn binding proteins in *B. henselae* WT and BadA^{-}. Bacteria were grown on hCBA and whole-cell bacteria were processed for mass spectrometry. DIA-MS was used for the detection of outer membrane proteins (OMP) previously described as Fn binding proteins (FnBPs) for *B. henselae* (2). **(A)** Omp43 (UniProt accession number A0A0R4J8D0); **(B)** Omp89 (UniProt accession number A0A0R4J719); and **c**, Pap31 (UniProt accession number A0A0R4J7W6). Note that all three proteins were expressed in both strains of *B. henselae* (WT and BadA^{-}) used in this study.

Accession	Coverage	#Peptides	#Unique
Proteins			
P02751-14|FINC_HUMAN	60%	83	83
P02751-10|FINC_HUMAN	58%	81	81
P02751-11|FINC_HUMAN	47%	75	75
P02751-12|FINC_HUMAN	55%	72	72
P02751-13|FINC_HUMAN	55%	80	80
P02751-16|FINC_HUMAN	47%	24	24
P02751-17|FINC_HUMAN	54%	81	81
P02751-1|FINC_HUMAN	52%	81	81
P02751-2|FINC_HUMAN	44%	22	22
P02751-3|FINC_HUMAN	55%	82	82
P02751-4|FINC_HUMAN	52%	69	69
P02751-5|FINC_HUMAN	53%	77	77
P02751-6|FINC_HUMAN	53%	76	76
P02751-7|FINC_HUMAN	52%	81	81
P02751-8|FINC_HUMAN	57%	82	82
P02751-9|FINC_HUMAN	59%	82	82
P02751|FINC_HUMAN	50%	80	80

Figure S8. Analysis of most prominent isoform in cellular Fn. DDA-MS analysis to identify the most prominent isoforms in *B. henselae* WT samples incubated with cellular Fn. The available isoform sequences for human Fn were retrieved from UniProt database (http://www.uniprot.org/)

Supplementary Tables

Table S1. Antibodies (IgG) and staining chemicals used in this study.

antibody	concentration	provider/reference
primary antibodies		
mouse anti-cellular fibronectin	1:100 (IF)	MAB1940, Sigma-Aldrich
mouse anti-fibronectin	1:10,000 (WB)	610077, Becton Dickinson
rabbit anti-fibronectin	1:2,000 (WB)	F3648, Sigma-Aldrich
rabbit anti-fibronectin	1:20 (IEM)	ab23750, Abcam
Alexa 488 conjugated rabbit anti-laminin	1:100 (IF)	NB300-144AF488, NOVUS
Alexa 647 conjugated rabbit anti-collagen V	1:100 (IF)	SC-166155AF647, Santa Cruz
rabbit anti-GAPDH	1:2,000 (WB)	PA1-16777, Thermo Fischer
rabbit anti-*B. henselae*	1:1,000 (ELISA); 1:1,1000 (IF)	(3)
rabbit anti-BadA	1:2,000 (WB)	(4)
secondary antibodies		
HRP conjugated anti-rabbit IgG	1:2,000 (ELISA); 1:1,000 (WB)	P0217, Dako
HRP conjugated anti-mouse IgG	1:1,000 (WB)	P0260, Dako
Alexa 488 conjugated anti-rabbit IgG	1:200 (IF)	111-545-045, Dianova
Alexa 488 conjugated anti-mouse IgG	1:400 (IF)	115-546-062, Dianova
Cy5 conjugated anti-mouse IgG	1:80 (IF)	115-175-062, Dianova
6 nm colloidal gold conjugated anti-rabbit IgG	1:40 (IEM)	111-195-144, Jackson ImmunoResearch
staining chemicals		
4',6-diamidino-2-phenylindole (DAPI)	1 µg/ml (IF)	1.24653, Merk
tetramethylrhodamine (TRITC) conjugated phalloidin	2.5 µg/ml (IF)	1951, Sigma-Aldrich

WB: Western blotting, IF: immunofluorescence microscopy; IEM: immunoelectron microscopy

Table S2. Oligonucleotides and plasmids used in this study.

oligonucleotides (guide RNAs)	sequence (5'-3')	references
gRNA_A	AAT CGG CGC GCG GTC GGC TG	this study
gRNA_B	GAC CGT CCC ATA TAA GCC C	this study
gRNA_C	CCG GGA GCC GGG GCT TAT AT	this study
gRNA_D	CAA GCG TGA GTA CTG ACC GC	this study
gRNA_E	CAA GCG TGA GTA CTG ACC G	this study
gRNA_F	AAT CTC TTC CTT ACT TGC GA	this study

plasmids	characteristics	references
lentiCRISPR v2	lentiviral transfer plasmid (no. 52961). Amp^r & Pur^r	Addgene
pMD2.G	envelope expressing plasmid (no. 12259), vesicular stomatitis virus G glycoprotein (VSV-G). Amp^r	Addgene
psPAX2	packaging plasmid (no. 12260). Amp^r	Addgene
pCR 2.1-TOPO vector	standard cloning vector for TA-overhangs. Kan^r & Amp^r	Invitrogen
pCR 2.1-TOPO_glyA	pCR 2.1-TOPO including the glyA fragment. Kan^r & Amp^r	this study
pCR 2.1-TOPO_hmbs	pCR 2.1-TOPO including the hmbs fragment. Kan^r & Amp^r	this study

PCR primers	oligonucleotide	sequence (5'-3')	annealing
Fn cDNA	fn_fwd<br>fn_rev	AAG ACC AGC AGA GGC ATA AG<br>AAA GCA CGA GTC ATC CGT AG	55°C
Actin cDNA	actin_fwd<br>actin_rev	AAA GAC CTG TAC GCC AAC AC<br>GTC ATA CTC CTG CTT GCT GAT	55°C
B. henselae<br>DNA quantification	glyA_fwd<br>glyA_rev	GAC AGG AAA ATG TGC CGA AT<br>GCA GGT GAA CCA AGA CGA AT	57°C
ECs<br>DNA quantification	hmbs_fwd<br>hmbs_rev	TTC CTT CCC TGA AGG GAT TCA CTC AG<br>TTA AGC CCA GCA GCC TAT CTG ACA CCC	57°C

Supplementary References

1. Szczesny P, Linke D, Ursinus A, Bär K, Schwarz H, Riess TM, Kempf VAJ, Lupas AN, Martin J, Zeth K. 2008. Structure of the head of the *Bartonella* adhesin BadA. PLoS Pathog 4:e1000119.

2. Dabo SM, Confer AW, Saliki JT, Anderson BE. 2006. Binding of *Bartonella henselae* to extracellular molecules: Identification of potential adhesins. Microb Pathog 41:10–20.

3. Kempf VAJ, Schaller M, Behrendt S, Volkmann B, Aepfelbacher M, Cakman I, Autenrieth IB. 2000. Interaction of *Bartonella henselae* with endothelial cells results in rapid bacterial rRNA synthesis and replication. Cell Microbiol 2:431–441.

4. Thibau A, Hipp K, Vaca DJ, Chowdhury S, Malmström J, Saragliadis A, Ballhorn W, Linke D, Kempf VAJ. 2022. Long-read sequencing reveals genetic adaptation of *Bartonella* Adhesin A among different *Bartonella henselae* isolates. Front Microbiol 13:1–17.
